# Evaluation of *Stemona collinsiae* root extracts for topical cockroach control: adulticidal, nymphicidal, and chemical distribution analysis

**DOI:** 10.1016/j.toxcx.2025.100225

**Published:** 2025-05-09

**Authors:** Aurapa Sakulpanich, Anon Phayakkaphon, Korawan Ounklong, Jinnaphat Sommanat, Yudthana Samung, Raweewan Srisawat, Jiraporn Ruangsittichai

**Affiliations:** a Division of Pharmaceutical Sciences, Faculty of Pharmacy, Thammasat University, Rangsit, Pathum Thani, 12120, Thailand; b Department of Medical Entomology, Faculty of Tropical Medicine, Mahidol University, Bangkok, 10400, Thailand; c National Science and Technology Development Agency, Thailand Science Park, Rangsit, Pathum Thani, 12120, Thailand

**Keywords:** *Stemona collinsae*, Non-tai-yak, Contact toxicity, Didehydrostemofoline, Alkaloids, Cockroach, Insecticide, MALDI IMS

## Abstract

*Stemona collinsiae* root extracts have been scientifically shown to exhibit antifeedant, growth inhibitory, larvicidal, pupacidal, and adulticidal activities in pests and insect vectors. In this research, contact toxicity of hexane, dichloromethane, ethanol, and water extracts was repeatedly tested on final-instar nymphs and adult *Periplaneta americana* using a topical application method and the penetration and distribution of didehydrostemofoline were detected at each of the specified times using MALDI-IMS and HPLC. Dichloromethane extract, which contained the highest didehydrostemofoline content, exhibited the highest contact toxicity against final-instar nymphs (41.0–100.0 % corrected mortality) and adult *Periplaneta americana* (23.0–46.0 % corrected mortality), while *P. americana* exposed to the water extract survived (0.0 % corrected mortality), similar to negative control group (0.0 % corrected mortality). Signs of toxicity such as excited movement, tremors, depression, motionlessness, expanded abdomen, and affected alimentary canal were observed in *P. americana* exposed to dichloromethane and hexane extracts. MALDI-IMS images showed that didehydrostemofoline was able to distribute from the sectioned first-abdominal segment to the sectioned head segment. HPLC chromatograms of the extracts of abdominal integument, lipid layer, alimentary canal, and head segment, it revealed that didehydrostemofoline initially adhered to the epicuticle, then penetrated through deeper layers of the integument and was distributed to other tissues. Therefore, *P. americana* could absorb didehydrostemofoline via topical administration, which was subsequently distributed and transported to other tissues. *S. collinsiae* dichloromethane root extract containing didehydrostemofoline could potentially be used as active ingredients in cockroach control.

## Highlights

• Dichloromethane extract of *Stemona collinsiae* roots contains the highest level of didehydrostemofoline and exhibits the most potent adulticidal and nymphicidal activities against *Periplaneta americana*.
• Didehydrostemofoline is penetrated and distributed in tissues detected by MALDI-IMS and HPLC analyses.
• Nymphal stages of *P. americana* exhibits evidently sensitivity to both dichloromethane and hexane extracts.

## Graphical abstract

**Figure:**
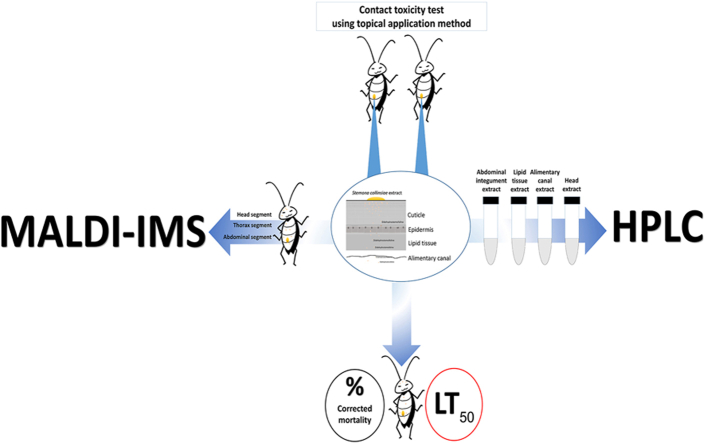

## 1 Introduction

The root of *Stemona collinsiae* Craib (Stemonaceae) has been used as an insecticide (Inthachub et al., 2010) and insect repellent for a long time in Thailand. Currently, the lipophilic extract of *S. collinsiae* root, along with didehydrostemofoline, has been scientifically shown to exhibit insecticidal and growth inhibitory activities, as well as contact toxicity against *Spodoptera littoralis* (Brem et al., 2002). Additionally, hydroxystemofoline (Phattharaphan et al., 2010) and 16,17-didehydro-16(E)-stemofoline (Jiwajinda et al., 2001), isolated from *S. collinsiae* root, has been found to inhibit the feeding of third-instar larvae of *Plutella xylostella*. Ethanolic extract of *S. collinsiae* root has demonstrated growth inhibitory activity against *Parasarcophaga ruficornis*, accelerating pupation in third-instar larvae such that mature adults could not emerge from irregularly shaped puparia (Sakulpanich et al., 2017). This effect was also observed in *Musca domestica* and *Chrysomya megacephala* (Sakulpanich et al., 2023). Furthermore, dichloromethane extract and hexane extract of *S. collinsiae* root, which contain didehydrostemofoline alkaloids and unknown fluorescent substances, have been shown to kill *Periplaneta americana* at both the final-instar nymph and adult stages via oral administration (Phayakkaphon et al., 2021). However, the contact toxicity of *S. collinsiae* root extracts against *P. americana*, a synanthropic insect and a significant vector with a strong survival ability, as well as the distribution of didehydrostemofoline within its tissues over time, should be analyzed. This research provides evidence of the contact toxicity of *S. collinsiae* root extracts on *P. americana* through topical administration and examines the distribution of didehydrostemofoline at various time points.

In this research, the nymphicidal and adulticidal activities of hexane, dichloromethane, ethanol, and water *S. collinsiae* root extracts against final-instar nymph and adult *P. americana* via contact administration was tested using a topical application method. The *S. collinsiae* extract solution was applied to the first segment of the abdominal sternites of *P. americana*, penetration and distribution of didehydrostemofoline were observed using modern technologies such as high performance liquid chromatography (HPLC) and matrix-assisted laser desorption/ionization imaging mass spectrometry (MALDI-IMS). These techniques, known for their high resolution and sensitivity, were utilized to detect the presence of specific substances and biological molecules within tissues. MALDI-IMS is convenient technique that allows the detection of substance distribution in different tissue regions without the need for phytochemical extraction. This technique involves a combination of thin tissue preparation, mass spectrometry, and microscopy, with data interpreted using specialized software. The interesting substance is ionized using MALDI, and analyte ions are detected by mass spectrometry. The resulting MS spectra, molecular ion images, and single-ion images of the compound of interest, along with optical images, were presented (Neumann et al., 2020; Spraker et al., 2020; Hanrieder et al., 2019; Stoeckli et al., 2001). For HPLC, didehydrostemofoline in dissected tissues was extracted using dichloromethane. Dichloromethane was used as extractant because didehydrostemofoline possessed a good solubility in dichloromethane (Phayakkaphon et al., 2021). Didehydrostemofoline was separated from the liquid tissue extract through HPLC column. Its presence in the liquid tissue extracts was detected by photodiode array detector. This experiment focused on didehydrostemofoline, an alkaloid identified as the major compound in *S. collinsiae* root and its insecticidal properties via oral (Brem et al., 2002; Phattharaphan et al., 2010; Jiwajinda et al., 2001; Phayakkaphon et al., 2021) and contact toxicity (Phattharaphan et al., 2010; Jiwajinda et al., 2001; Sakulpanich et al., 2017, 2023). The study aimed to investigate the potential contact toxicity and penetration of didehydrostemofoline through the exoskeleton into deeper tissues of *P. americana*. MALDI-IMS and HPLC were employed to detect the penetration and distribution of didehydrostemofoline within *P. americana* tissue at various time points. These findings may contribute to the development of a contact insecticide product containing natural *S. collinsiae* root extract, potentially leading to the creation of liquid or spray or aerosol formulations.

## 2 Methodology

### 2.1 Plant materials

*Stemona collinsiae* roots were collected from Ubon Ratchathani, Thailand on December 2018 and January 2019. Whole plant with root of *S. collinsiae* was identified and deposited in the Forest and Plant Conservation research office, Department of National Parks, Wildlife and Plant Conservation with voucher specimens BKF No. 196976. The roots were cleaned with tap water. The roots were dried with electric fan until surface water was eliminated. The roots were cut into small pieces. Then, the roots were dried in hot air oven at 55 °C for 72 h. Dried roots were ground with rotor mill (Retsch Model SK300, Retsch GmbH, Germany). Powdered root was used in further experiment.

### 2.2 Chemicals and reagents

All organic solvents and reagents were analytical grade. Water type I, produced by Milli-Q®, Germany, was used in this experiment.

### 2.3 Preparation of extracts (modified from Phayakkaphon et al., 2021)

The powdered root was sequentially extracted using a reflux extraction method with hexane, dichloromethane, ethanol, and water as solvents. Initially, hexane (300 mL) was added to a round-bottom flask containing the powdered root (250 g). The flask was placed in a warm water bath, with the temperature maintained at 70 °C, and fitted with a cooling condenser. The extraction was performed continuously for 1 h. The liquid extract was then filtered through Whatman™ 1 filter paper (CAT No. 1001-240, Global Life Sciences Solution Operations UK Ltd., England), and the filtrate was collected in a light-protected glass bottle. Hexane was added to a residue of *S. collinsiae.* The process of hexane extraction was repeatedly done until the alkaloids were exhaustively extracted. The completion of the extraction was verified using thin-layer chromatography (TLC). TLC was conducted on a silica gel GF_254_ plate (Merck, Germany) using a mobile phase composed of dichloromethane, ethyl acetate, methanol, and 10 % NH_4_OH in a ratio of 70:25:5:1. Dragendorff's spray reagent was used to detect alkaloids, with the presence of an orange band indicating their presence.

Following the hexane extraction, the residue was dried to remove any residual hexane, and the extraction process was repeatedly performed using dichloromethane (300 mL) instead of hexane. The extraction with dichloromethane was carried out using the same procedure as with extraction using hexane. After completing the extraction with dichloromethane, the dichloromethane was replaced with ethanol, and the extraction was conducted as before. Finally, ethanol was replaced with water for the last extraction.

liquid hexane, dichloromethane and ethanol extract were concentrated using a rotary evaporator and dried in a water bath at 70 °C, except for the water extract, which was concentrated and dried by lyophilization. This process yielded crude extracts of hexane, dichloromethane, ethanol, and water. The chemical constituents of the four extracts were detected using the TLC method.

### 2.4 Detection of chemical constituent in the four extracts with TLC method

TLC was carried on a silica gel GF_254_ TLC plate (Merck, Germany), with a mobile phase consisting of dichloromethane, ethyl acetate, methanol, and 10 % NH_4_OH in a ratio of 70:25:5:1. The extract solutions and didehydrostemofoline reference substance were spotted by Linomat 5 (Camag®, Switzerland). The developed TLC plate was imaged by TLC visualizer 3 (Camag®, Switzerland). The spotting, image visualization and documentation were controlled by HPTLC software visionCATS (Camag®, Switzerland). Dragendorff's spray reagent was used to detect alkaloids, with the presence of an orange band indicating the presence of alkaloids.

### 2.5 Ethical consideration statement

The protocol was provided under ethical principles and guidelines for the use of animals provided by the National Research Council of Thailand. All experiments abide by protocols which was approved by the Animal care and Use Committee of Thammasat University: Protocol No. 005/2020 and the Animal care and Use Committee of Faculty of Tropical Medicine, Mahidol University: Protocol No. 001/2021 Certificate No. FTM-ACUC 005/2021.

### 2.6 *Periplaneta americana* rearing

*Periplaneta americana* was collected from Ratchaburi, Thailand. The species was identified by Yudthana Samung, an entomologist. The species identification was performed following the procedures outlined in the Handbook of Domiciliary Cockroach Species in Thailand (Asahina, 1983). *P. americana* were reared by Anon Phayakkaphon at the Faculty of Tropical Medicine, Mahidol University. *P. americana* were fed dry cat food (Purina® Friskies® for adult cats, Nestlé, Thailand) and provided with water. They were kept in plastic boxes (30 × 30 × 30 cm) with lids, and petroleum jelly was applied to the inside walls of the boxes to prevent the cockroaches from escaping. The boxes were placed in a cockroach-rearing room with ambient temperatures of 27–30 °C, humidity levels of 70 %–90 %, and a 12:12 h dark-light cycle.

In this experiment, final-instar nymphs (5–6 months old, 3.0–3.5 cm in length) and adult cockroaches (7–10 months old, 3.0–4.0 cm in length) were used. The weight ranges of the tested final-instar nymphs and adult *P. americana* were 0.8–1.5 g and 1.2–2.5 g, respectively. Selection of *P. americana* for contact toxicity testing, All *P. americana* specimens had intact organs and a normally colored exoskeleton. Individuals with missing limbs, wings, antennae, or other physical damage were excluded. The selected specimens exhibited normal movement, good sensitivity, and a strong defensive response.

### 2.7 Contact toxicity of the four extracts using topical application method (modified from Syed et al., 2013; Saenmanot et al., 2018; Sakulpanich et al., 2021)

#### 2.7.1 Sample preparations

Hexane and dichloromethane extracts were dissolved in acetone, while ethanol and water extracts were dissolved in 70 % ethanol. Complete dissolution of the extracts was achieved using sonication in an ultrasonic bath. The four extracts were then diluted, and a series of two-fold dilutions were prepared, ranging in concentration from 1.0 % to 12.0 % w/v (g/mL), for contact toxicity testing. All *P. americana* in the negative control group were exposed to acetone only. The positive control group was treated with a solution of synthetic pesticide imidacloprid in acetone, with concentrations ranging from 0.025 % to 6.0 % w/v (g/mL) for contact toxicity testing. The percentage of corrected mortality was calculated. The range of extract concentrations and imidacloprid concentration that produced the percentage of corrected mortality between 10 and 90 % (Finney, 1971) against *P. americana* were used to calculate the LC_50_.

#### *2.7.2* *Contact toxicity test using topical application method* (modified from Syed et al., 2013)

Final-instar nymphs (10 unsexed nymphs, 5–6 months old, 3.0–3.5 cm in length, 0.8–1.5 g in weight) and adult cockroaches (10 cockroaches, mixed sexes, 7–10 months old, 3.0–4.0 cm in length, 1.2–2.5 g in weight) were anesthetized using a chilling method to prevent interference from other chemicals. They were placed in a freezer at −20 °C for 10–15 min. The chilling process of *P. americana* was frequently monitored to avoid any fatalities. A 20 μL volume of each extract solution was applied to the first segment of the abdominal sternites of each *P. americana* using a 100 μL syringe (Camag®, Switzerland). The same technique was used for the negative control group, which received only acetone, and the positive control group, which received imidacloprid solution. All groups of *P. americana* were kept at ambient temperatures (27–30 °C), with 70 %–90 % humidity, and a photoperiod of 12:12 h dark cycle. Mortality of final-instar nymphs and adults *P. americana* was observed at 1, 2, 6, 12, 24, 48, 72, 96, and 120 h after treatment. Death of *P. americana* was determined by prodding with forceps. Signs of death included no response to prodding, lack of mobility, and inability to return to a normal posture. Signs of toxicity were also observed and recorded. All experiments were performed in triplicate.

### 2.8 Detection of external appearance and dissection of dead *P. americana* in the extract-treated groups comparing with negative and positive control groups

Images of the bodies of deceased and euthanized *P. americana* were taken photo. The bodies were dissected using medical scissors and pins. Each *P. americana* was pinned with pins on a dissection board. The abdomen was longitudinally cut through the thorax using medical scissors. The exoskeleton and fat tissue were carefully removed with forceps. The dissected alimentary canal, along with the head, was separated. The alimentary canals from the extract-treated group were compared with those from the negative control group and the positive control group. Photos of the dissected alimentary canals were taken using a Canon EOS 500D digital SLR camera (Canon, Japan). A SNZ745T stereomicroscope (10x) (Nikon, China) with an MDX503 microscope camera and iWorks software (Lanoptik Technologies Ltd., China) was used to magnify and capture the images.

### 2.9 Detection of median lethal time (LT_50_) at three specific concentrations

Three specific concentrations (0.01 % w/v, 1.5 % w/v, and 10 % w/v) were tested in final-instar nymphs and adult *P. americana*. Each solution of hexane, dichloromethane, ethanol, and water extracts was applied to the first segment of the abdominal sternites of final-instar nymphs (10 unsexed nymphs) and adult *P. americana* (10 mixed-sex cockroaches). Mortality was recorded at 1, 2, 4, 6, 24, 48, 72, 96, and 120 h. The results for each extract were compared with those from a negative control group treated with acetone only and a positive control group treated with an imidacloprid solution. LT_50_ values were calculated using Probit analysis, as described in the Parameters and Statistical Analysis section. All experiments were performed in triplicate.

The toxicity of the hexane and dichloromethane extracts was directly related to their concentration. The 0.01 % w/v concentration was approximately 100 times lower than the lowest tested concentration (1 % w/v). This concentration was used because some final-instar nymph showed signs of toxicity after they were exposed with the dichloromethane crude extract. The effects of extracts at a concentration of 0.01 % w/v against *P. americana* were assessed, and the LT_50_ value for this low concentration was determined. The concentration of 1.5 % w/v was selected because it represented the LC_50_ value of the dichloromethane extract observed in the final-instar nymph group. A maximum concentration of 12 % w/v was not tested in LT_50_ detection or in the detection of penetration and distribution of didehydrostemofoline, as it caused severe damage to the alimentary canal in final-instar nymphs and some adult *P. americana*. Moreover, cryosectioning to detect the penetration and distribution of didehydrostemofoline could not be performed at this concentration (12 %w/v). Instead, a lower concentration of 10 % w/v was used. Therefore, the three specific concentrations (0.01 %, 1.5 %, and 10 % w/v) were chosen for the detection of LT_50_, onset of action, and penetration and distribution of didehydrostemofoline.

### 2.10 Detection of penetration and distribution of didehydrostemofoline using MALDI-IMS method

The MALDI-IMS (Matrix-Assisted Laser Desorption/Ionization Imaging Mass Spectrometry) technique is known for its high sensitivity, selectivity, and resolution. It allows for the direct detection of didehydrostemofoline within the tissue of *P. americana* without the need for phytochemical extraction. In this study, didehydrostemofoline was used as a chemical marker and detected using MALDI-IMS. In this experiment, dichloromethane and water crude extracts were used to study the penetration and distribution of didehydrostemofoline. The dichloromethane extract was chosen because it contained the highest concentration of didehydrostemofoline and exhibited the strongest nymphicidal and adulticidal activities. In contrast, didehydrostemofoline was not found in the water extract, and no significant biological activities were observed.

The separated segments of *P. americana* were sectioned, and the tissue sections were coated with an appropriate MALDI matrix. This matrix facilitates the desorption and ionization of didehydrostemofoline within the tissue. Upon laser irradiation, the ionization of didehydrostemofoline occurred, and the ionized molecules of didehydrostemofoline were detected using a mass spectrometer. The resulting data were used to generate images showing the distribution and intensity of the ionized didehydrostemofoline in the sectioned tissues. These results were presented as IMS images, optical images, and overlay images, which revealed the penetration and distribution of didehydrostemofoline in the tissue.

#### 2.10.1 Preparation of didehydrostemofoline reference substance

A solution of didehydrostemofoline in dichloromethane at a concentration of 0.2 mg/mL was prepared and analyzed using MALDI-IMS qualitative analysis. The mass spectra, molecular mass, and fragment pattern of the didehydrostemofoline reference substance were collected and utilized for the detection of didehydrostemofoline in *P. americana* tissue in subsequent experiments.

#### 2.10.2 Detection of didehydrostemofoline distribution in *P. americana* after treatment with dichloromethane and water crude extracts

*P. americana* were divided into six groups, with each group consisting of 10 cockroaches. The first, second, and third groups received a dichloromethane extract solution and were observed for 15, 30, and 120 min, respectively. The fourth, fifth, and sixth groups received a water extract solution and were observed for the same time intervals.

Solutions of the dichloromethane and water extracts were applied to the first segment of the abdominal sternites of *P. americana*. At 15, 30, and 120 min post-treatment, three *P. americana* specimens were sampled from each group. They were euthanized by freezing at −20 °C for 2 h. The specimens were then sectioned, and the penetration and distribution of didehydrostemofoline were analyzed using the MALDI-IMS method.

#### 2.10.3 Preparation of the tissue and cryosectioning

Due to the large body size of *P. americana*, its entire body could not be fixed on a single slide. Therefore, the specimen was divided into three sections: head, thorax, and abdomen. Each section was embedded in optimal cutting temperature (OCT) compound (FSC 22 Blue Frozen Section Compound, Leica, USA) and cryosectioned at 50 μm using a cryostat microtome (CM1950, Leica, Germany). The sectioned tissues were then mounted on indium tin oxide (ITO) coated glass slides (Sigma Aldrich, St. Louis, United States).

#### 2.10.4 Coating section with matrix

The sectioned tissues, mounted on ITO slides, were automatically sprayed with α-Cyano-4-hydroxycinnamic acid (CHCA) (Sigma Aldrich, Darmstadt, Germany) using the iMLayer (Shimadzu, Japan). The film thickness was controlled at 0.7 μm, with a deposition time of 8 min and a deposition temperature maintained at 250 °C.

#### 2.10.5 MALDI IMS analysis

Imaging mass spectrometry (IMS) was conducted using the iMScope TRIO (Shimadzu, Japan) equipped with a laser-diode-excited Nd (YAG laser) and MALDI-ESI ionization method was used for qualitative analysis. The MS acquisition parameters and positive mode of ion polarity, with a pitch of 37 μm and a molecular mass range of 382–390 m/z were set. The sample voltage was set at 3.00 kV, and the detector voltage was set at 2.00 kV. The laser firing parameters were configured to 100 shots at a repetition rate of 1000 Hz, with the laser diameter and intensity fixed at 2 and 20.0, respectively.

#### 2.10.6 Image processing and data analysis

Image processing was carried out using Imaging MS Solution version 1.30 software (Shimadzu, Japan). Data analysis was performed with ACD Labs 2018 to search for mass (m/z) chemical structures within the PubChem and ChemSpider databases (>90 million structures). The MS spectra, molecular mass, and fragment pattern of detectable didehydrostemofoline in the tissue were compared with the didehydrostemofoline reference substance.

### 2.11 Detection of penetration and distribution of didehydrostemofoline using HPLC method

Initially, the detection of didehydrostemofoline in the abdominal integument, lipid tissue, alimentary canal, and head extracts was performed using the TLC method. After spraying with Dragendorff's reagent, a pale orange color appeared on the track of the abdominal integument extract, indicating the presence of the didehydrostemofoline alkaloid, as confirmed by comparison with the didehydrostemofoline reference substance. However, didehydrostemofoline was not detectable in the lipid tissue, alimentary canal, or head extracts, likely due to its very low concentrations in these tissues. Therefore, the detection of didehydrostemofoline in each tissue extract was further analyzed using the HPLC method, instead of the TLC method, with comparisons made to the didehydrostemofoline reference substance and *S. collinsiae* extracts.

From the contact toxicity test and chemical constituent analysis of *S. collinsiae* extracts using the TLC method, the dichloromethane extract exhibited the highest content of didehydrostemofoline and demonstrated the strongest nymphicidal and adulticidal activities. The testing procedure was explained as follows.

#### 2.11.1 Collection of abdominal integument, lipid tissue, alimentary canal, and head from *P. americana* exposed to S. collinsiae extract at different exposure times

*P. americana* were divided into eight treatment groups (10 cockroaches/groups) based on collection times: 0.25, 0.5, 1, 2, 4, 6, 12, and 24 h, with a negative control group receiving only acetone. Solutions of dichloromethane crude extract at concentrations of 1.5 % and 10 % w/v, in volumes of 20 μL, were applied to the first segment of the abdominal sternites of *P. americana*. At each of the specified times (0.25, 0.5, 1, 2, 4, 6, 12, and 24 h), all live *P. americana* were euthanized by freezing at −20 °C for 2 h. The abdominal integument where the solution was applied, lipid tissue, alimentary canal, and head were separately dissected. Each tissue was placed in individual glass test tubes with screw caps for didehydrostermofoline extraction.

#### 2.11.2 Preparation of abdominal integument, lipid tissue, alimentary canal and head extracts

Extracts from the abdominal integument, lipid tissue, alimentary canal, and head segments were prepared using dichloromethane as the extractant due to its effectiveness in dissolving didehydrostemofoline. To each glass test tube with a screw cap containing dissected tissue, 1 mL of dichloromethane was added. The containers were sonicated in a sonication bath for 5 min. The liquid dichloromethane extract was then transferred to a new glass test tube with a screw cap. An additional 1 mL of dichloromethane was added to the original glass test tube with a screw cap containing the dissected tissue, followed by another 5-min sonication. This extraction process was repeated three times. For the head extract, the head segment was ground before being subjected to the same extraction procedure. The collected liquid dichloromethane extracts were then evaporated using a rotary evaporator under reduced pressure at 40 °C, resulting in a dried residue. The dried residue was added with the 1 mL volume of dichloromethane and it was mixed thoroughly until homogeneous. The solution was then filtered using a 0.45 μm nylon syringe filter. The presence of didehydrostemofoline in each tissue extract solution was subsequently detected using HPLC.

#### 2.11.3 HPLC apparatus and conditions (modified from Kongkiatpaiboon et al., 2012)

HPLC was conducted using a Shimadzu Technologies modular Class VP system, which included an SCL-20A system controller, a UV–vis SPD-20A detector, an LC-20AD pump, and an SIL-20A auto-injector (Shimadzu, Japan). An isocratic elution was employed. A BDS Hypersil™ C18 column (150 mm × 4.6 mm I.D., 5 μm particle size) (Lot: 11065, Thermo Fisher Scientific, Inc.) with a C18 guard column was used for separation. The mobile phase was a mixture of methanol and 1 mM ammonium acetate in a 55:45 ratio. The injection volume for each *P. americana* extract was 20 μL. Injection volumes for the didehydrostemofoline reference substance solution (0. 2 mg/mL) were 5 μL. Injection volumes for the dichloromethane crude extract (10 mg/mL) were 2 μL. The optimized flow rate was set at 1 mL/min. Didehydrostemofoline absorbance was detected at 295 nm. This wavelength was suitable for detection of didehydrostemofoline. The total analytical run time was 30 min. Retention times of didehydrostemofoline in the tissue extracts were determined and compared with the retention times of the didehydrostemofoline reference substance and didehydrostemofoline in the dichloromethane crude extract.

### 2.12 Parameters and statistical analysis

The percentage of observed mortality was calculated, and the percentages of corrected mortality were adjusted using Abbott's formula (Abbott, 1925). Signs of toxicity, behavior, and morphological changes were observed and recorded. Time-to-event was calculated using survival analysis (IBM SPSS Statistics version 28.0.0, IBM Corporation, USA). *P. americana* specimens receiving different *S. collinsiae* crude extracts were divided into groups based on the severity level of signs of toxicity and symptoms leading to death, including the type of extract. Median lethal concentrations (LC_50_) and median lethal time (LT_50_) were analyzed using the Probit analysis program (March 1987 version) (Raymond, 1985) and IBM SPSS Statistics version 28.0.0 (IBM Corporation, USA) with 95 % confidence limits (UCL and LCL). Results with a *p*-value <0.05 were considered statistically significant. Results of the experiment were presented as a range of minimum and maximum values and mean ± SD.

## 3 Results

### 3.1 Chemical constituent in the four extracts

**Fig. 1:**
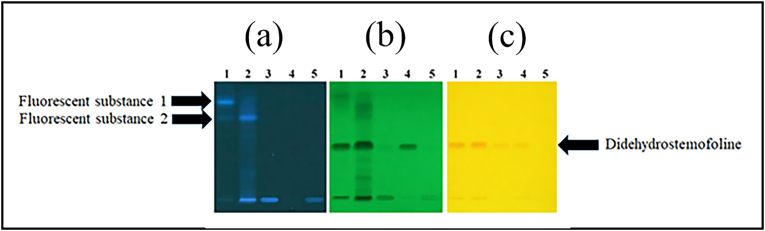
TLC analysis of tested crude extracts under different visualization conditions, compared with didehydrostemofoline. (a) Fluorescent bands observed under UV light (365 nm): fluorescent substance 1 appeared as a bright blue band in the upper section of TLC plate while fluorescent substance 2 appeared as a slightly less intense blue band below fluorescent substance 1, (b) quenching bands observed under UV light (254 nm): didehydrostemofoline was clearly detected in the dichloromethane crude extract as the darkest and thickest quenching band. (c) Visualization after derivatization with Dragendorff's spray reagent: didehydrostemofoline appeared as a distinct orange band at the same relative position across tracks. Tracks 1–5 exhibited variations in the number of bands and color intensity, indicating differences in chemical composition and didehydrostemofoline content among the extracts. Track 1 = hexane crude extract, Track 2 = dichloromethane crude extract, Track 3 = ethanol crude extract, Track 4 = didehydrostemofoline reference substance, Track 5 = water crude extract. (For interpretation of the references to color in this figure legend, the reader is referred to the Web version of this article.)

Chemical constituents and didehydrostemofoline in hexane, dichloromethane, ethanol and water crude extracts were detected by using TLC method. Difference of chemical constituent in each extract was showed in this experiment. Didehydrostemofoline was clearly found in hexane (**Track No. 1 in** Fig. 1b and c), dichloromethane (**Track No. 2 in** Fig. 1b and c), and ethanol (**Track No. 3 in** Fig. 1b and c) crude extracts at R_f_ value of 0.41, comparing with didehydrostemofoline reference substance (**Track No. 4 in** Fig. 1b and c). Under UV light at 254 and 366 nm, dichloromethane crude extract (**Track No. 2 in** Fig. 1a and b) exhibited chemical compositions more than hexane crude extract (**Track No. 1 in** Fig. 1a and b) and ethanol crude extract (**Track No. 3 in** Fig. 1a and b). At the starting line of TLC plate, the high intensity of quenching band was found in the dichloromethane crude extract more than hexane, ethanol and water crude extracts. Under UV light at 254 nm, several dark quenching bands and didehydrostemofoline band were prominently presented in the dichloromethane crude extract (**Track No. 2 in** Fig. 1b). At the same concentration and under UV light at 366 nm, fluorescence substance 1 and 2 were found in both hexane (**Track No. 1 in** Fig. 1a) and dichloromethane crude extracts (**Track No. 2 in** Fig. 1a). Fluorescent substance 1 highly displayed in the hexane crude extract (**Track No. 1 in** Fig. 1a). Fluorescence substances 2 showed a high intensity in the dichloromethane crude extract (**Track No. 2 in** Fig. 1a) compared to the hexane crude extract (**Track No. 1 in** Fig. 1a). Fluorescence substances 1 and 2 were not found in ethanol crude extract (**Track No. 3 in** Fig. 1a). Neither fluorescent substances 1 and 2 nor didehydrostemofoline were found in the water crude extract (**Track No. 5 in** Fig. 1a–1c). Under UV light at 254 nm and after spray TLC plate with Dragendorff's spray reagent, the highest content of didehydrostemofoline showed in the dichloromethane crude extract (**Track No. 2 in** Fig. 1b and c). In the water crude extract, the higher polarity substances remained at the baseline (starting line) under both UV light at 254 and 366 nm (**Track No. 5 in** Fig. 1a and b).

### 3.2 Contact toxicity, signs of toxicity, and LC_50_ of the four extracts

In a concentration range of 1.0–12.0 % w/v, the highest percentage of corrected mortality was observed in final-instar nymphs (41.0–100.0 %) and adult *P. americana* (23.0–46.0 %) when treated with dichloromethane crude extract (Table 1). The second-highest mortality was observed in final-instar nymphs (43.0–83.0 %) and adult *P. americana* (17.0–43.0 %) exposed to hexane crude extract. Both stages of *P. americana* exposed to ethanol and water crude extracts showed no mortality (0.0 ± 0.0 % corrected mortality) (Table 1). In comparison, *P. americana* treated with imidacloprid at concentrations ranging from 0.025 to 6.0 % w/v exhibited a corrected mortality percentage of 16.7–100.0 %. The LC_50_ values were calculated based on the concentrations of the extracts and imidacloprid that produced corrected mortality between 10 and 90 %. The LC_50_ values at 48 h, for final-instar nymphs exposed to dichloromethane and hexane crude extracts were determined to be 1.5 ± 0.2 % w/v and 2.1 ± 0.3 % w/v, respectively (Table 1). The LC_50_ value of imidacloprid was calculated as 0.13 ± 0.01 % w/v. Comparably, contact toxicity of hexane, dichloromethane, ethanol, and water crude extracts against *P. americana*, it was found that the hexane extract and dichloromethane extract greatly exhibited nymphicidal activity, with the dichloromethane extract being particularly effective. In contrast, the ethanol and water extracts did not show any nymphicidal and adulticidal activities (Table 1).

**Table 1:** Percentage of corrected mortality, LC_50_, LT_50_ and onset time of signs toxicity presenting in the both stages of *P. americana*.

	Concentration	Final-instar nymph				
	(%w/v)	Hexane	Dichloromethane	Ethanol	Water	Negative control
% corrected mortality	1–12 % w/v	43.0–83.0	41.0–100.0	0.0–0.0	0.0–0.0	0.0–0.0
at 48 h		(17.0–43.0)	(23.0–46.0)	(0.0–0.0)	(0.0–0.0)	(0.0–0.0)
LC_50_ at 48 h (%w/v)		2.1 ± 0.3	1.5 ± 0.2	ND	ND	ND
		(ND)	(ND)	(ND)	(ND)	(ND)
LT_50_ (hours)	0.01	ND	ND	ND	ND	ND
		(ND)	(ND)	(ND)	(ND)	(ND)
	1.5	ND	47.3 ± 6.8	ND	ND	ND
		(ND)	(97.5 ± 8.5)	(ND)	(ND)	(ND)
	10	48.0 ± 9.2	36.1 ± 0.8	ND	ND	ND
		(79.7 ± 19.5)	(62.2 ± 2.9)	(ND)	(ND)	(ND)
Onset of excited state (hours)	0.01	ND	ND	ND	ND	ND
		(ND)	(ND)	(ND)	(ND)	(ND)
	1.5	ND	ND	ND	ND	ND
		(ND)	(0.4 ± 0.02)	(ND)	(ND)	(ND)
	10	ND	0.5 ± 0.01	ND	ND	ND
		(3.3 ± 0.01)	(0.5 ± 0.03)	(ND)	(ND)	(ND)
Onset of immobility (hours)	0.01	ND	ND	ND	ND	ND
		(ND)	(ND)	(ND)	(ND)	(ND)
	1.5	ND	ND	ND	ND	ND
		(43.3 ± 8.6)	(29.4 ± 8.7)	(ND)	(ND)	(ND)
	10	2.0 ± 1.0	1.6 ± 0.1	ND	ND	ND
		(6.0 ± 0.01)	(1.7 ± 0.2)	(ND)	(ND)	(ND)
Onset of expanded abdomen (hours)	0.01	ND	ND	ND	ND	ND
		(ND)	(ND)	(ND)	(ND)	(ND)
	1.5	ND	15.1 ± 4.2	ND	ND	ND
		(48.5 ± 0.01)	(27.8 ± 0.6)	(ND)	(ND)	(ND)
	10	3.0 ± 1.0	1.6 ± 0.1	ND	ND	ND
		(23.9 ± 0.2)	(15.5 ± 7.1)	(ND)	(ND)	(ND)

(…) = Results of adult *P. americana*.

ND = Not detectable.

The same signs of toxicity, including excited movement, hind leg shaking, whole-body tremors, immobility, and abdominal swelling, were observed in the group of *P. americana* exposed to hexane, dichloromethane and ethanol crude extracts. Irreversible and severe abdominal distension, leading to death, was frequently observed in *P. americana* exposed to the dichloromethane crude extract. Signs of toxicity, such as depression and motionlessness, predominantly occurred in *P. americana* exposed to the ethanol crude extract. Notably, the expanded abdomen observed in this group could revert to normal. No signs of toxicity were observed in *P. americana* exposed to the water crude extract. All *P. americana* in this group exhibited a defensive response, characterized by rapid locomotor movement and heightened sensitivity to stimuli.

*P. americana* treated with imidacloprid solution at concentrations ranging from 0.025 % to 6.0 % w/v exhibited symptoms such as tremors, rapid locomotion, severe ventral abdominal contractions, knockdown with tremors, and swollen abdomen, ultimately leading to death. The signs of toxicity occurred in group of *P. americana* exposed to imidaclopride solution and the extracts were similar. But, the severe ventral abdominal contractions were absent in the group treated with dichloromethane and hexane crude extracts.

### 3.3 Median lethal time (LT_50_) and onset of actions at three specific concentrations of the four extracts

At a concentration of 0.01 % w/v, hexane, dichloromethane, ethanol, and water crude extracts were not able to kill all *P. americana* individuals. However, only dichloromethane extract caused very mild signs of toxicity in some final-instar nymphs, including body elevation, hind leg scratching of the abdomen, and very weakly body shaking with short period. These symptoms eventually disappeared. But, onset of excited state was not clear. It was recorded as not detectable. At the same concentration (0.01 % w/v), *P. americana* treated with imidacloprid solution exhibited signs of toxicity, such as mild body shaking, motionlessness, and a lack of response to stimuli, which also eventually disappeared. Ultimately, at this concentration, all *P. americana* survived (0 % corrected mortality) (Table 1).

At an *S. collinsiae* extract concentration of 1.5 % w/v, only the dichloromethane extract was able to kill final-instar nymphs and adult *P. americana*, with LT_50_ values of 47.3 ± 6.8 and 97.5 ± 8.5 h, respectively. At this concentration of 1.5 % w/v, adult *P. americana* dropped with dichloromethane extract exhibited an excited state at 0.4 ± 0.02 h, immobility at 29.4 ± 8.7 h, and expanded abdomen at 27.8 ± 0.6 h, respectively while final instar nymph exhibited expanded abdomen at 15.1 ± 4.2 h, respectively. When the extract concentration was increased to 10 % w/v, the dichloromethane crude extract showed even more potent nymphicidal and adulticidal activities, with LT_50_ values of 36.1 ± 0.8 and 62.2 ± 2.9 h, respectively. The hexane crude extract demonstrated nymphicidal and adulticidal activities, with LT_50_ values of 48.0 ± 9.2 and 79.7 ± 19.5 h, respectively (Table 1).

At the high concentration of 10 % w/v dichloromethane crude extract solution, all signs of toxicity were observed in both final-instar nymphs and adult *P. americana*. The onset of the excited state occurred in final-instar nymphs at 0.5 ± 0.01 h and in adult *P. americana* at 0.5 ± 0.03 h, followed by immobility at 1.6 ± 0.1 h and 1.7 ± 0.2 h for final-instar nymphs and adult *P. americana*, respectively. The onset of a swollen abdomen in final-instar nymphs and adult *P. americana* occurred at 1.6 ± 0.1 and 15.5 ± 7.1 h, respectively. At concentrations of 1.5 % and 10 % w/v, the onset of signs of toxicity in adult *P. americana* such as immobility and expanded abdomen generally occurred later than in final-instar nymphs. Consequently, final-instar nymphs were more sensitive to hexane and dichloromethane crude extracts than adult *P. americana*. At concentrations of 0.01 %, 0.5 %, and 10 % w/v, and across all treatment durations, the ethanol and water crude extract of *S. collinsiae* were unable to eliminate all *P. americana* (Table 1), similar to the negative control group receiving only acetone. These individuals survived and showed no signs of toxicity due to the absence of insecticidal phytochemicals (Fig. 1). Dichloromethane crude extract caused signs of toxicity and mortality of *P. americana* more quietly than the hexane crude extract, observed from onset of actions and LT_50_ values. However, toxicity symptoms in *P. americana* exposed to imidacloprid appeared more rapidly than in those exposed to the dichloromethane and hexane crude extracts.

### 3.4 External appearances and dissection of dead *P. americana*

**Fig. 2:**
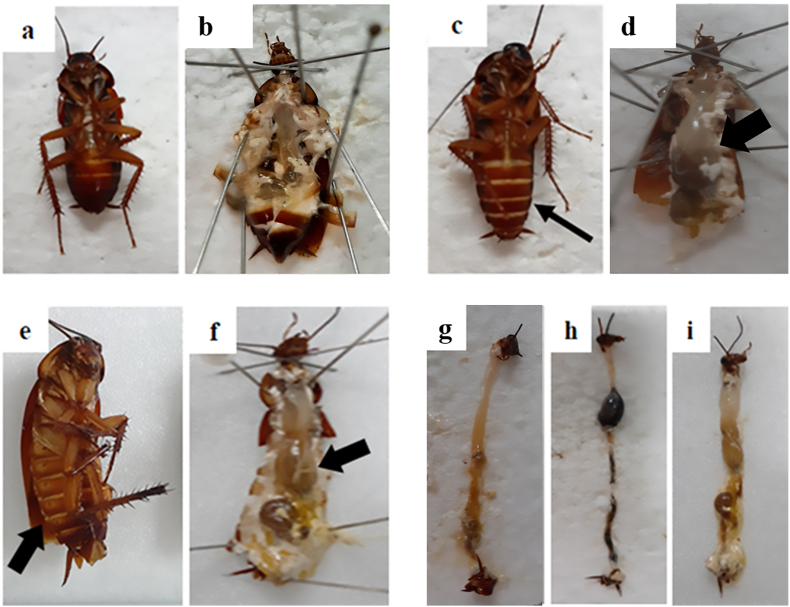
Comparison of (a) *P. americana* treated with acetone only before dissected, (b) dissected *P. americana* receiving acetone only showing normal fore gut, (c) predominant swollen abdomen part of *P. americana* after dropping solution of dichloromethane crude extract (black arrow), (d) a dissected body of *P. americana* receiving solution of dichloromethane crude extract displaying an expanded fore gut of alimentary canal (black arrow), (e) *P. americana* receiving imidaclopride solution showing extended abdomen (black arrow), (f) dissected *P. americana* treated with imidaclopride solution presenting swollen fore gut (black arrow), (g) dissected alimentary canal of *P. americana* receiving acetone (h) dissected alimentary canal of *P. americana* receiving dichloromethane crude extract and (i) dissected alimentary canal of *P. americana* receiving imidaclopride solution.

The appearance of an expanded abdomen was observed in *P. americana* exposed to hexane crude extract, dichloromethane crude extract, and imidacloprid solution. Predominantly expanded abdomens were especially evident in *P. americana* treated with the dichloromethane crude extract solution (Fig. 2c, **black arrow**). Similarly, a swollen abdomen was observed in the positive control group of *P. americana* exposed to the imidacloprid solution (Fig. 2e, **black arrow**). Swollen abdomens were also seen in some *P. americana* treated with the solution of ethanol crude extract, but this symptom was not consistently presented. In contrast, *P. americana* treated with the water crude extract did not exhibit expanded abdomens, similar to the negative control group treated with acetone only (Fig. 2a). Upon dissection of the bodies of deceased and euthanized *P. americana*, the alimentary canals at the foregut of *P. americana* exposed to the dichloromethane crude extract solution (Fig. 2d and h) and the imidacloprid solution (Fig. 2f and i) appeared swollen, resembling a bubble. This swelling of the alimentary canal was not observed in the *P. americana* treated with water crude extract, which was similar to the negative control group treated with acetone (Fig. 2b and g).

Comparing between four extracts, the highest severity of expanded abdomen and swollen alimentary canal was found in group of *P. americana* exposing dichloromethane crude extract and followed by *P. americana* contacting with hexane crude extract.

### 3.5 Distribution of didehydrostemofoline in tissue of *P. americana* detecting with MALDI- IMS

**Fig. 3:**
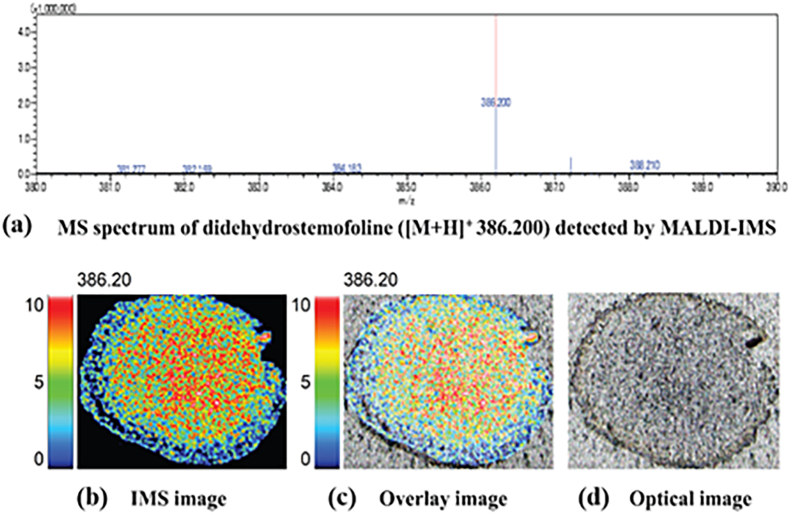
Detection of didehydrostemofoline reference substance with MALDI-IMS method showed (a) IMS spectrum, (b) IMS image, (c) overlay image and (d) optical image.

Initially, a reference substance, didehydrostemofoline ([M+H]^+^ 386.200), was detected. It could be identified using MALDI-IMS. The MS spectrum of didehydrostemofoline was shown in Fig. 3a. In the IMS images, didehydrostemofoline was represented by blue, green, and red spots based on its intensity (Fig. 3b). Additionally, overlay images (Fig. 3c) and optical images (Fig. 3d) were produced.

**Fig. 4:**
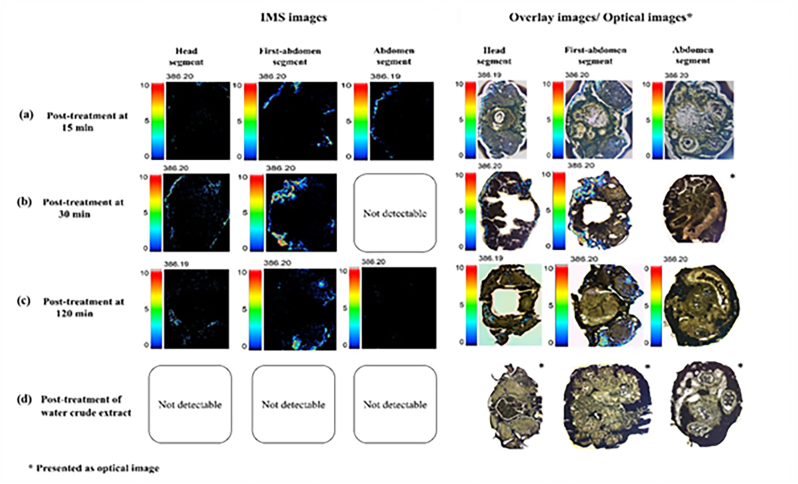
IMS images exhibited the distribution of didehydrostemofoline in adult *P. americana* exposed to the dichloromethane crude extract solution at (a) 15 min, (b) 30 min, and (c) 120 min. Didehydrostemofoline was not detected in (d) *P. americana* exposed to the water crude extract solution.

Didehydrostmofoline in the sectioned tissues of euthanized *P. americana* exposing with the dichloromethane extract were detectable using MALDI-IMS. It found that at 15 min post-treatment (Fig. 4a), the highest intensity of blue and green spots, representing didehydrostemofoline, was observed in the sectioned tissue of the first abdominal segment (Fig. 4a, **first-abdomen segment**), followed by a border of abdomen segment (Fig. 4a, **abdomen segment**) and a sectioned head part (Fig. 4a, **head segment**). A lower intensity of spots was found in the sectioned abdominal tissue (Fig. 4a, **abdomen segment**) due to errors in tissue border and spacing during sectioning. At 30 min, the intensity of the spots in the sectioned tissue of the first abdominal segment (Fig. 4b, first-abdomen segment) and head segment increased significantly (Fig. 4b, **head segment**). They were more widely and deeply distributed in the first abdominal segment (Fig. 4b, **first-abdomen segmen**t). The spots also increasingly distributed in the border and central of the sectioned head part (Fig. 4b, **head segment**) while no spots were observed in the sectioned abdominal part, as shown in the optical image (Fig. 4b, **abdomen segment**). By 120 min (Fig. 4c), the spots were scattered throughout the first abdominal segment and head segment of adult *P. americana* exposed to the dichloromethane crude extract, with a smaller intensity of spots found in the abdominal segment. The distribution of didehydrostemofoline appeared in the IMS images of euthanized *P. americana* exposed to the dichloromethane crude extract solution (Fig. 4a–c), but it was not found in the tissue of euthanized *P. americana* treated with the water crude extract solution, as shown in the optical images in Fig. 4d.

### 3.6 Distribution of didehydrostemofoline in tissue of *P. americana* detecting with HPLC

**Fig. 5:**
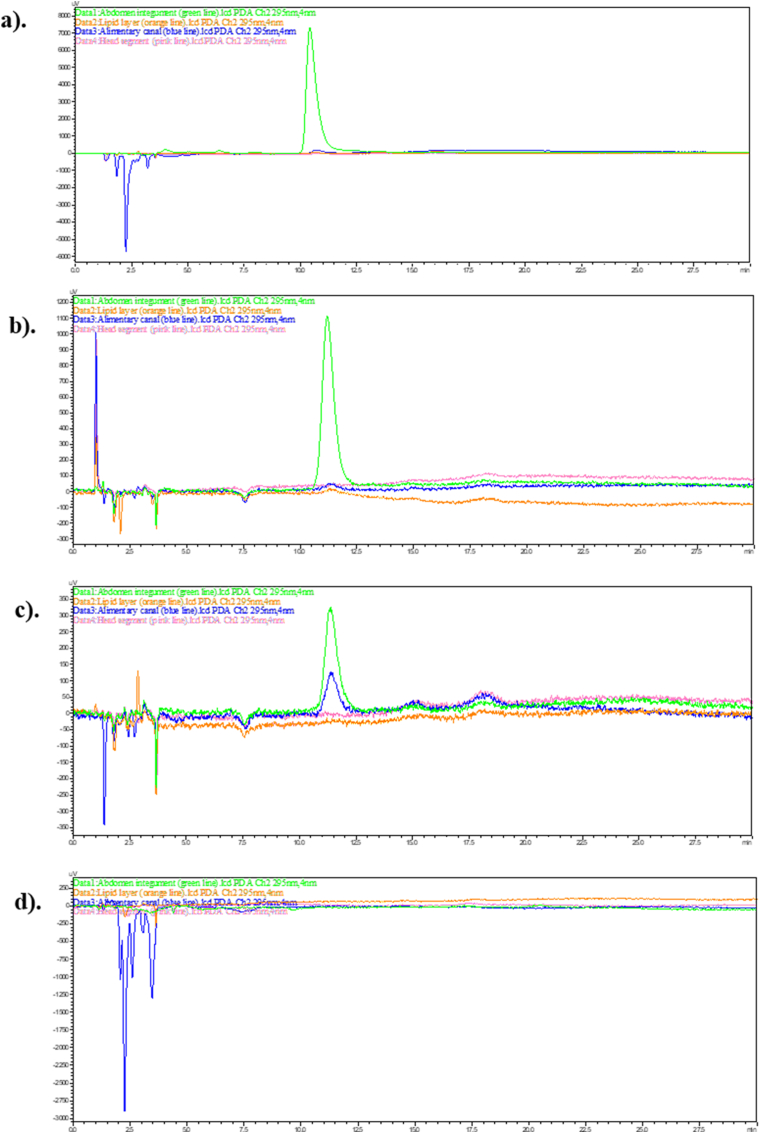
HPLC chromatograms of didehydrostemofoline appearing in the abdomen integument (green line), lipid layer (orange line), alimentary canal (blue line) and head extracts (pink line), after adult *P. americana* dropped with 1.5 % w/v dichloromethane crude extract at (a) 15 min, (b) 2 h, (c) 24 h, comparing with (d) adult *P. americana* in negative control group receiving acetone only which did not appear a peak of didehydrostemofoline. (For interpretation of the references to color in this figure legend, the reader is referred to the Web version of this article.)

Since the highest concentration of didehydrostemofoline was clearly appeared in dichloromethane extract. Thus, dichloromethane extract was used in this experiment. Two concentrations (1.5 % and 10 % w/v) of the dichloromethane crude extract were tested, and the results were compared. After treating *P. americana* with a 1.5 % w/v solution of the dichloromethane crude extract for 0.25–24 h (Fig. 5), didehydrostemofoline was detected in all abdominal integument extracts. The height of didehydrostemofoline peak in each time decreased respectively. It showed that the content of didehydrostemofoline in the abdominal integument extracts subsequently decreased (Fig. 5a–c, **green line**). However, didehydrostemofoline was not clearly detectable in the lipid tissue (Fig. 5a–c, **orange line**) and head extracts (Fig. 5a–c, **pink line**) due to the presence of very small amounts. At 24 h, very low content of didehydrostemofoline was identified in the alimentary canal extract (Fig. 5c, **blue line**).

**Fig. 6:**
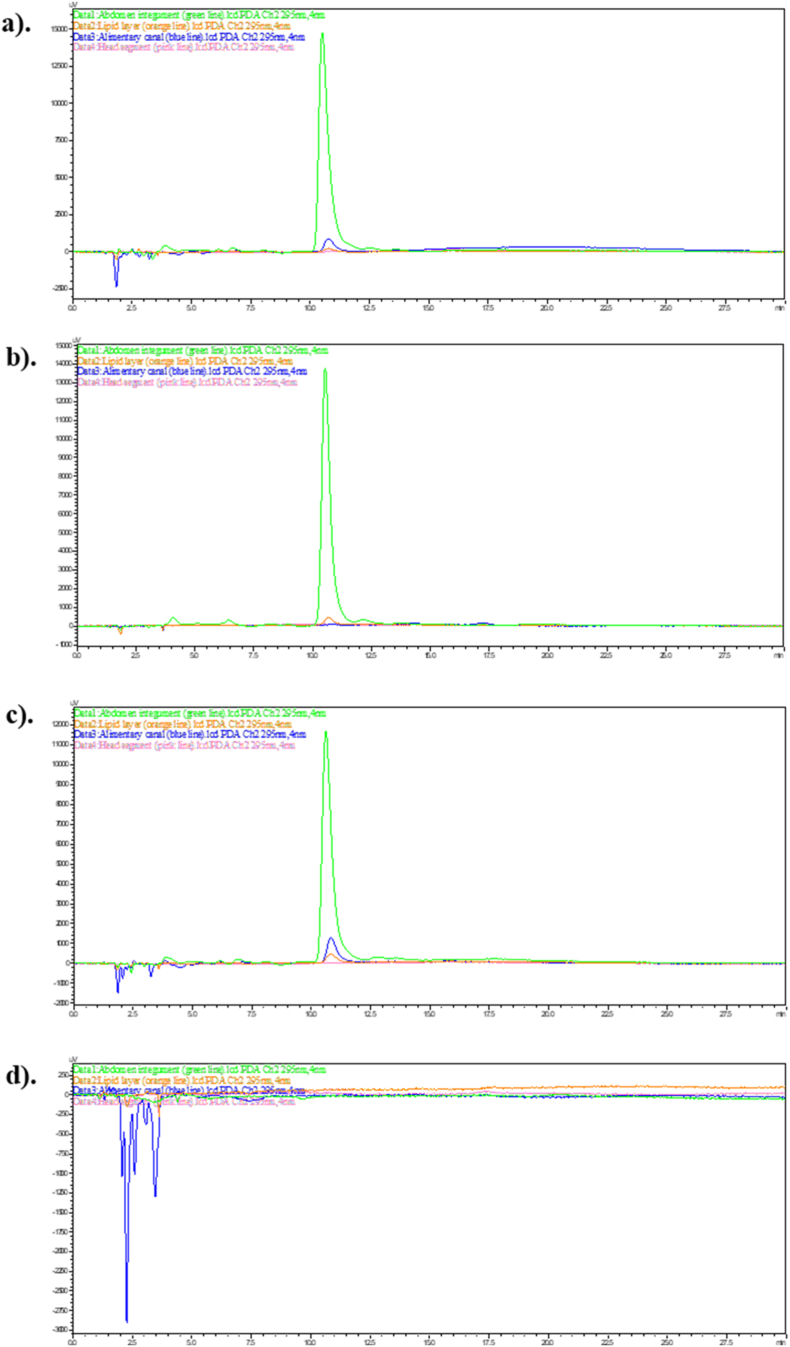
HPLC chromatograms of didehydrostemofoline appearing in the abdomen integument (green line), lipid layer (orange line), alimentary canal (blue line) and head extracts (pink line), after adult *P. americana* dropped with 10 % w/v dichloromethane crude extract at (a) 15 min, (b) 2 h, (c) 24 h, comparing with (d) adult *P. americana* in negative control group receiving acetone only which did not appear a peak of didehydrostemofoline. (For interpretation of the references to color in this figure legend, the reader is referred to the Web version of this article.)

When the concentration of dichloromethane extract was increased to 10 % w/v, didehydrostemofoline was still present in the abdominal integument extracts (Fig. 6a–c, **green line**) in all times. Additionally, at 24 h, didehydrostemofoline was clearly detected in the lipid tissue (Fig. 6c, **orange line**), alimentary canal (Fig. 6c, **blue line**), and head extracts (Fig. 6c, **pink line**). From HPLC chromatograms of abdominal integument extract in each time (Fig. 6a–c, green line), the height of didehydrostemofoline peak decreased respectively. It showed that the content of didehydrostemofoline was decreased. Didehydrostemofoline could be penetrated and absorbed in tissues. In contrast, didehydrostemofoline was absent in all *P. americana* specimens in the negative control group, which received only acetone (Fig. 6d).

**Fig. 7:**
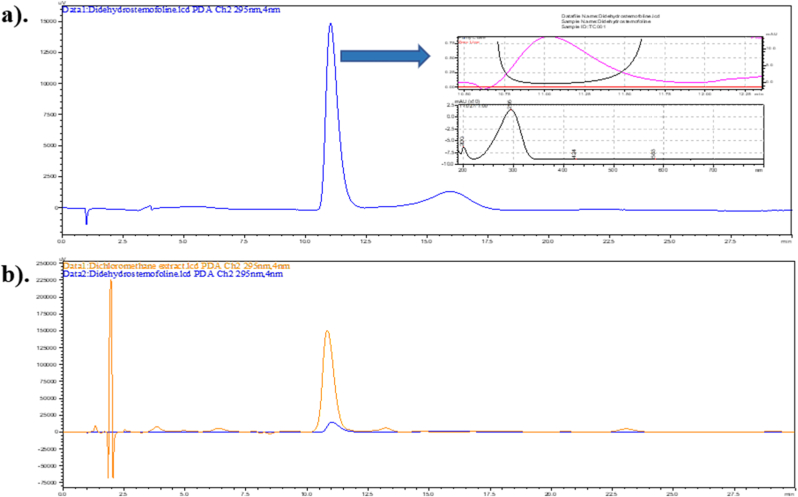
HPLC chromatograms of a). didehydrostemofoline reference substance and b). didehydrostemofoline in dichloromethane crude extract comparing with didehydrostemofoline reference substance, detected at 295 nm.

After *P. americana* exposing with 5 % and 10 % w/v of dichloromethane crude extract, the retention time of didehydrostemofoline in each tissue extract was at 10.95 ± 0.29 min, closely matching that of the didehydrostemofoline reference substance presenting at 10.92 ± 0.08 min (Fig. 7a) and the didehydrostemofoline in the dichloromethane crude extract, which exhibited a retention time of 10.87 ± 0.06 min (Fig. 7b).

**Fig. 8:**
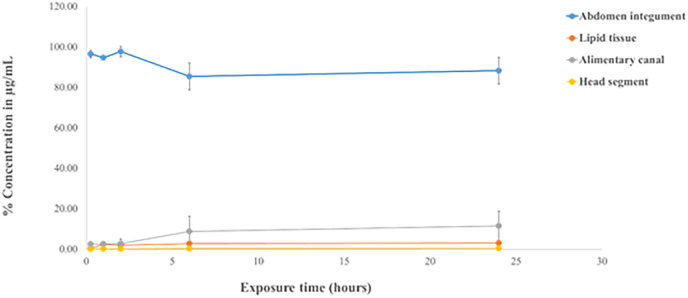
Concentration of didehydrostemofoline in extracts of abdomen integument, lipid tissue, alimentary canal and head segment, extracted from adult *P. americana*, at different exposure time.

Following post-treatment with a 10 % w/v dichloromethane crude extract solution for 0.25–24 h, the content of didehydrostemofoline in the abdominal integument gradually decreased from 96.53 ± 1.81 % to 88.30 ± 6.65 % (Fig. 8, **blue line**). In contrast, didehydrostemofoline levels in the lipid tissue (Fig. 8, **orange line**) and alimentary canal (Fig. 8, **gray line**) increased from 0.52 ± 0.24 % to 3.17 ± 0.50 % and from 2.54 ± 1.33 % to 11.53 ± 7.43 %, respectively. Additionally, the didehydrostemofoline content in the head extract showed a slight increase from 0.09 ± 0.03 % to 0.53 ± 0.26 % (Fig. 8, **yellow line**). As the concentration of the dichloromethane crude extract and exposure time increased, the penetration and distribution of didehydrostemofoline in the lipid tissue, alimentary canal, and head regions also increased accordingly (Fig. 8)

## 4 Discussion

*Periplaneta americana* is a synanthropic insect, insect vector, and nuisance pest (Li et al., 2024; Zheng et al., 2022). It transmits pathogenic microorganisms to animals and humans via mechanical and biological routes (Khodabandeh et al., 2020; Donkor, 2020). Serious infectious diseases such as typhoid (Donkor, 2020), shigellosis (Donkor, 2020), and parasitic diseases (Akeju et al., 2024; Falsone et al., 2017) have been associated with *P. americana.* Allergens derived from *P. americana* can also induce cockroach allergies (Pomés and Arruda, 2023). Furthermore, *P. americana* is an omnivorous and opportunistic feeder, consuming various foods such as rice, grains, cheese, bananas, and meat. Agricultural products are at risk of destruction and microbial contamination due to these pests. The presence of cockroaches indicates poor sanitation and can lead to non-compliance with Good Manufacturing Practices (U.S. Food and Drug Administration, 2024). Thus, effective cockroach control is used for disrupting their life cycle, reducing cockroach populations and the incidence of associated infectious and non-infectious diseases. In Thailand, biopesticidal plants are used for pest control, with *S. collinsiae* root being among them. Indigenous Thais traditionally used *S. collinsiae* root in pepper plantations to protect plants from pests (Inthachub et al., 2010). Recent studies have shown that extracts from *S. collinsiae* roots and its isolated compounds as stemofoline and didehydrostemofoline effectively kill neonate larvae of *Spodoptera littoralis* via contact route (Brem et al., 2002). This raises the question of whether *S. collinsiae* could also be effective against *P. americana* though contact route*.* Thus, this study aimed to evaluate the contact toxicity of *S. collinsiae* root extract against *P. americana*. Crude extracts of *S. collinsiae* roots were used in this experiment, because crude extract containing high content of active ingredients and exhibiting high strength of insecticidal activity can be prepared easily through simple processes. Using reflux extraction method with four types of extractants, four distinct extracts—hexane, dichloromethane, ethanol, and water crude extracts—were obtained. From this contact toxicity experiment, the dichloromethane crude extract demonstrated the highest nymphicidal and adulticidal activities against *P. americana*, followed by the hexane crude extract. Both the dichloromethane and hexane crude extracts obviously contained didehydrostemofoline and unknown fluorescent substances. The key difference between the both extracts was the content of didehydrostemofoline, unknown fluorescent substances and several other compounds. The dichloromethane crude extract had the highest concentration of didehydrostemofoline, along with some blue fluorescent substances (fluorescent substance 2) and dark quenching substances. Additionally, the dichloromethane crude extract displayed more compounds than the other crude extracts, as shown in TLC chromatograms (Fig. 1). The hexane crude extract contained a lower concentration of didehydrostemofoline but clearly exhibited blue fluorescent substances I. In contrast, the water crude extract contained neither didehydrostemofoline nor blue fluorescent substances. Upon testing with four extracts via contact route, signs of toxicity such as excited movement, hind leg tremors, generalized tremors, immobility, and abdominal distension were observed in the group of *P. americana* exposed to hexane, dichloromethane, and ethanol crude extracts of *S. collinsiae*. After dissection, swelling of the alimentary canal in the abdominal region was consistently observed in groups of *P. americana* that received dichloromethane and hexane crude extracts. The severity of the toxicity signs varied by extract. The severity of the toxicity was categorized into three levels based on observed symptoms, onset of action, signs of impending death, and the concentrations of the extracts, including their insecticidal compounds. The dichloromethane crude extract exhibited the highest severity level, correlating with its highest concentration of didehydrostemofoline, blue fluorescent substances 2 and various phytochemicals. Irreversible and severe abdominal distension, often leading to mortality, was frequently observed in *P. americana* exposed to the dichloromethane crude extract. The second severity level, classified as mild, was observed with the ethanol crude extract, which contained a very low concentration of didehydrostemofoline and dark quenching compounds but lacked blue fluorescent substances. More polar compound was at starting line (Fig. 1). Mild signs of toxicity, such as depression and motionlessness, predominantly occurred in *P. americana* exposed to the ethanol extract. Notably, the expanded abdomen in this group could revert to normal, allowing all *P. americana* to survive. The third severity level, categorized as non-toxic, was found with the water crude extract. No signs of toxicity were observed in *P. americana* exposed to the water extract and the negative control group receiving only acetone, as it did not contain didehydrostemofoline, fluorescent substances I and II, or any insecticidal substances. Polar compounds were found at starting line. All *P. americana* in this group exhibited a defensive response characterized by rapid locomotor movement and heightened sensitivity to stimuli. The water extract did not exhibit any nymphicidal or adulticidal activities against *P. americana*. All *P. americana* survived. Compared to oral toxicity tests (Phayakkaphon et al., 2021), all *P. americana* that consumed bait containing the water crude extract survived without exhibiting any signs of toxicity. *P. americana* consuming bait mixed with the ethanol crude extract displayed weak signs of toxicity, which eventually resolved. However, a small number of *P. americana* were killed after consuming bait containing the ethanol extract (Phayakkaphon et al., 2021). In terms of contact toxicity, the residual didehydrostemofoline in the ethanol crude extract caused only temporary signs of toxicity and did not result in mortality (0.0 % corrected mortality) because the solution of the ethanol crude extract was applied only once to the first segment of the abdominal sternites of *P. americana*. The concentration of didehydrostemofoline in the ethanol crude extract was insufficient to eliminate *P. americana*. Interestingly, both oral toxicity (Phayakkaphon et al., 2021) and contact toxicity of the crude extracts exhibited the same signs of toxicity; however, the strength of toxicity differed. In contact toxicity, tearing of the exoskeleton and leakage of adipose tissue between arthrodial membranes were not observed in *P. americana* exposed to a single application of the dichloromethane crude extract solution. Only one severe sign of toxicity, a protruded anus, was observed after *P. americana* was exposed to a high concentration of dichloromethane crude extract (12 % w/v). However, more severe signs of toxicity may occur in *P. americana* exposed to higher concentrations of the dichloromethane crude extract solution or with repeated exposure. Thus, the potency of nymphicidal and adulticidal activities against *P. americana*, including the strength of toxicity signs, was directly related to the types and concentrations of insecticidal chemical constituents in *S. collinsiae* extracts, while the onset of action was indirectly related. Nymph *P. americana* exposed to solution of dichloromethane and hexane crude extract was easily and rapidly killed more than adult *P. americana*. Considering positive control, imidacloprid was used in contact toxicity test. It revealed that imidacloprid induced signs of toxicity, including tremors, rapid uncoordinated movement, abdominal contractions, irreversible abdominal distension, and ultimately the death of *P. americana.* The signs of toxicity in *P. americana* exposed to imidacloprid solution were similar to those seen in *P. americana* exposed to the dichloromethane and hexane crude extracts. However, the potency of imidacloprid (LC_50_ value of 0.13 ± 0.01 % w/v) was higher than that of the dichloromethane (LC_50_ value of 1.5 ± 0.2 % w/v) and hexane extracts (LC_50_ value of 2.1 ± 0.3 % w/v). Compared to the previous study (Phayakkaphon et al., 2021), imidacloprid in the toxic bait caused signs of toxicity similar to those observed in *P. americana* exposed to the imidacloprid solution and *P. americana* exposed to dichloromethane and hexane crude extract solution. The similarity of expressed signs of toxicity of imidacloprid and the crude extracts arises because the active phytochemicals or toxicophores in their chemical structure were the same, interacted with the same type of receptor and exhibited the same signs of toxicity. Imidacloprid, a synthetic insecticide that mimics nicotine alkaloids, belongs to the neonicotinoid class. Its chemical structure includes chloronicotinyl and nitroamino-imidazoline functional groups, which interact with nicotinic acetylcholine receptors (Buszewski et al., 2019), provoking cholinergic signaling that leads to neurological symptoms such as tremors, ataxia, and reduced activity. Notably, the ethanol extract contained a small amount of didehydrostemofoline and absence of the other substances, it showed mild signs of toxicity. Additionally, didehydrostemofoline has been scientifically proven to possess contact toxicity against *Spodoptera littoralis* (Brem et al., 2002). Therefore, the observed signs of toxicity in *P. americana* exposed to the hexane, dichloromethane, and ethanol crude extracts may be attributed to the presence of didehydrostemofoline in these extracts. However, the specific toxicophores of didehydrostemofoline that interact with nicotinic acetylcholine receptors remain unclear. Further experiments are needed to investigate these toxicophores and the didehydrostemofoline-receptor complex. Additionally, fluorescent substance 1 and 2, didehydrostemofoline and lipophilic compounds in hexane and dichloromethane crude extract promoted nymphicidal and adulticidal activities against *P. americana*. The good activities may cause form mixture of these compounds in the hexane and dichloromethane crude extract. Consequently, didehydrostemofoline in the dichloromethane crude extract is of interest for studying its penetration and distribution in adult *P. americana* tissue using MALDI-IMS and HPLC.

Didehydrostemofoline was able to be penetrated, distributed and transported to other parts. From IMS image, the distribution of didehydrostemofoline was primarily concentrated in the first abdominal segment, particularly at the surface area of the integument. It was also slightly present in the head segment. By 30 min, the didehydrostemofoline had increasingly distributed into deeper layers of both the first abdominal and head segments. At 120 min, didehydrostemofoline was predominantly found in the first abdominal segment, head segment and abdomen segment. Didehydrostemofoline was possibly transported to other parts based on the circulation of hemolymph (Gullan, 2014; Bell, 1982). The first abdominal integument, where the dichloromethane crude extract was applied, served as the entry point for didehydrostemofoline. The didehydrostemofoline likely penetrated and permeated through the integument, diffusing into the hemocoel and eventually reaching the head. It accumulated in nerve tissue, as shown in IMS images of the sectioned head segment between 15 and 120 min. Thus, it is likely that didehydrostemofoline was transported to the head via the hemolymph route and accumulated in nerve and ganglia tissue. However, longitudinal section of euthanized *P. americana* exposing with dichloromethane extract should be also performed. Transportation of didehydrostemofoline was more obviously seen. But size of *P. americana* was bigger than slide. Thus, transverse section of *P. ameircana* was done in this experiment. Revisiting the IMS images of the sectioned head segment at 15 min, a small amount of didehydrostemofoline was detected at the border or surface of the head integument. By 30 min, the penetration of didehydrostemofoline had increased at the border or surface of the head integument. This penetration might have occurred due to lateral movement after the insecticide diffused from the cuticles into the epidermal layers, with lateral movement occurring at the interface between the endocuticle and epidermal layers (Gerolt, 1983). Transporting didehydrostemofoline to its site of action could occur through various pathways, which should be further validated through experiments. In addition to the IMS method, HPLC was used as a second method for the detection of didehydrostemofoline in tissue extracts. When didehydrostemofoline penetrates through integument and tissue layers, it would be found in the tissue extracts. In the group of *P. americana* exposed to the 10 % dichloromethane crude extract, didehydrostemofoline was obviously detected in the tissue extracts of abdominal integument, lipid layer, alimentary canal, and head segment, indicating its accumulation in these tissues. However, in the group of *P. americana* exposed to the 1.5 % dichloromethane crude extract, didehydrostemofoline was not clearly detected in the lipid tissue and head segment because didehydrostemofoline content in the 1.5 % w/v of the dichloromethane crude extract had approximately 7–10 times less than the 10 % w/v of the extract. This suggests that the penetration of didehydrostemofoline through the integument is directly related to the concentration of the dichloromethane crude extract applied to the first abdominal segment.

HPLC chromatograms and IMS images confirm that a high concentration of didehydrostemofoline attaches to the epicuticle and gradually diffuses to deeper layers of the cuticle based on the concentration gradient. Additionally, the physicochemical properties of didehydrostemofoline influence its penetration, absorption, and distribution within tissues. Didehydrostemofoline (National Library of Medicine, 2023) is composed of six nitrogen and oxygen atoms, has a molecular weight of less than 500 Da, and exhibits lipophilic properties with a calculated alogP of 2.4. It has a hydrogen bond donor count of 0, a hydrogen bond acceptor count of 6, a rotatable bond count of 3, a formal charge of 0, and a topological polar surface area (TPSA) of 57.2 Å^2^. Considering that effective insecticides typically possess properties such as a molecular weight between 150 and 500, mlogP between 0 and 5, alogP between 0 and 6.5, 1-8 H-bond acceptors, ≤2 H-bond donors, and ≤2 rotatable bonds (Tice, 2001), didehydrostemofoline is likely to be transported across cell membranes. It may also present good permeability via diffusion (Denecke et al., 2018; Webb and Green, 1945). Based on the physicochemical properties described and HPLC results, which showed significant adherence of didehydrostemofoline to the epicuticle and its gradual penetration to deeper layers and tissue distribution, it is plausible that didehydrostemofoline could cross cell membranes based on the diffusion effect and Fick's law (Tice, 2001; Pradhan et al., 1952; Webb and Green, 1945). The diffusion effect drives the penetration and permeation of insecticides across the epicuticle and integument, depending on the concentration gradient between the epicuticle (high concentration area) and other layers (low concentration area). For example, dust mixed with pure insecticides attaches to the epicuticle, and the high concentration of lipophilic insecticides in the dust is diffused to layers with lower concentrations. Given the epicuticle's composition, which includes cuticular lipids (Gullan, 2014), and didehydrostemofoline's lipophilic, unionized small molecule characteristics, didehydrostemofoline adheres to and accumulates in cuticular lipids. This accumulation supports good diffusion, similar to how DDT, due to its lipophilic properties, accumulates in cuticular wax and then diffuses effectively (Pradhan et al., 1952). Moreover, didehydrostemofoline possibly was penetrated from the epicuticle and permeate through the procuticle, epithelium, and lipid layers via a transcellular route. The cuticles of *P. americana* include dermal gland ducts (Scheie et al., 1968), wax canal filaments, and pore canals with diameters of approximately 0.15 μm (Webb and Green, 1945), and are rich in lipophilic substances. Due to the small molecule size, zero net charge, and lipophilicity of didehydrostemofoline, it is likely to dissolve and diffuse via appendageal routes and may traverse each epithelium cell through intercellular spaces. However, further experiments are required to confirm this. Furthermore, other insecticidal compounds were reported that they were found in *S. collinsiae* such as stemofoline (MW 387.5) (Brem et al., 2002), hydroxystemofoline (MW 403.5) (Phattharaphan et al., 2010), 2′-hydroxystemofoline, stemona acetal, stemonal (MW 372.3) (Shiengthong et al., 1974), and stemonone (MW 370.3) (Shiengthong et al., 1974). Also, stemofoline possessed contact toxicity (Brem et al., 2002). It is a pity that these compounds were not tested in this experiment. These compounds may have penetrated and been distributed to other tissues. For example, rotenone was able to penetrate through integuments and exhibited contact toxicity against cockroaches (Davidson et al., 1930). It should be tested in the further experiment.

Furthermore, the issue of eliminating adult *P. americana* using dichloromethane crude extract of *S. collinsiae* via contact application was found to be dependent on the concentration used. The extract should be applied at concentrations higher than 12 % w/v to achieve complete mortality of adult *P. americana*. When concentrations lower than 12 % w/v were used, all *P. americana* were not completely killed. They were able to repeatedly come into contact with the extract. This repeated exposure could reduce the susceptibility of adult *P. americana* to the extract, potentially leading to insecticide resistance. Such resistance could negatively impact the effectiveness of *P. americana* control. Therefore, ensuring the use of an effective concentration of dichloromethane crude extract, didehydrostemofoline, and other insecticidal compounds is crucial for effective cockroach control. Testing for *P. americana* resistance to the dichloromethane crude extract and didehydrostemofoline, as well as conducting through evaluations of pesticide risks in mammals, should be prioritized in future research.

Summarily, in this research, the dichloromethane crude extract of *S. collinsiae* roots demonstrated significant nymphicidal and adulticidal activities when administered topically. Didehydrostemofoline, a key compound, could penetrate and distribute to other tissues. Didehydrostemofoline and *S. collinsiae* root extract were related to the stimulation of acetylcholine neurotransmitters and the inhibition of acetylcholinesterase (Sastraruji et al., 2010). Therefore, the dichloromethane *S. collinsiae* root extract containing didehydrostemofoline alkaloids possesses insecticidal activity comparable to that of imidacloprid which is classified in group of neonicotinoid insecticides and possess nicotinic acetylcholine receptor competitive modulators (Buszewski et al., 2019). Dichloromethane *S. collinsiae* root extract containing didehydrostemofoline alkaloids have potential as active ingredients in cockroach control, particularly in liquid or aerosol or spray formulations for future applications, similar to a contact insecticide product containing imidacloprid.

## 5 Conclusion

*Stemona collinsiae* root extracts have demonstrated bioinsecticidal properties, effectively disrupting the life cycle of *P. americana*. This experiment aimed at development of insecticidal plant extract containing active ingredient. The dichloromethane extract of *S. collinsiae*, which contains the highest concentration of didehydrostermofoline and blue fluorescent substance, is particularly lethal to *P. americana* when applied topically. The final instar nymph stage was identified as the most susceptible, with a majority being killed by the dichloromethane crude extract. Signs of toxicity in *P. americana* exposed to dichloromethane and hexane crude extracts of *S. collinsiae* included leg shaking, tremors, depression, immobility, swollen abdomen, and enlarged foregut. These symptoms closely resemble those observed in *P. americana* that exposed with imidaclopride solution and consumed toxic bait mixed with the crude extracts. This similarity is likely due to the presence of the same active compounds and toxicophores interacting with the identical target sites or receptor. Increasing the concentration of the dichloromethane crude extract enhanced its insecticidal potency, leading to quicker elimination of *P. americana*. Didehydrostermofoline in the dichloromethane crude extract is capable of diffusing through the integument, after which it is distributed to lipid tissues and possibly transported to the alimentary canal and head segment. The compound accumulates in nerve tissue, which may contribute to its insecticidal effects. Given these properties, the dichloromethane crude extract of *S. collinsiae* root has potential for development as an active ingredient in liquid and aerosol formulations. However, determining the effective concentrations needed to eliminate both nymphal stages and the more tolerant adult *P. americana* remains a challenge in the development of liquid, aerosol and spray formulations.

## CRediT authorship contribution statement

**Aurapa Sakulpanich:** Writing – review & editing, Writing – original draft, Visualization, Validation, Software, Resources, Project administration, Methodology, Investigation, Funding acquisition, Formal analysis, Conceptualization. **Anon Phayakkaphon:** Validation, Resources, Investigation. **Korawan Ounklong:** Writing – original draft, Visualization, Validation, Software, Resources, Investigation, Formal analysis. **Jinnaphat Sommanat:** Resources, Investigation. **Yudthana Samung:** Resources, Investigation. **Raweewan Srisawat:** Software, Resources. **Jiraporn Ruangsittichai:** Writing – original draft, Resources, Project administration.

## Ethical statement

Hereby, I, Aurapa Sakulpanich, consciously assure that for the manuscript Evaluation of *Stemona collinsiae* root extracts for topical cockroach control: adulticidal, nymphicidal, and chemical distribution analysis the following is fulfilled.

1) This material is the authors' own original work, which has not been previously published elsewhere.
2) The paper is not currently being considered for publication elsewhere.
3) The paper reflects the authors' own research and analysis in a truthful and complete manner.
4) The paper properly credits the meaningful contributions of co-authors and co-researchers.
5) The results are appropriately placed in the context of prior and existing research.
6) All sources used are properly disclosed (correct citation). Literally copying of text must be indicated assuch by using quotation marks and giving proper reference.
7) All authors have been personally and actively involved in substantial work leading to the paper, andwill take public responsibility for its content.

The violation of the Ethical Statement rules may result in severe consequences.

To verify originality, your article may be checked by the originality detection software iThenticate. See also http://www.elsevier.com/editors/plagdetect.

I agree with the above statements and declare that this submission follows the policies of Solid State.

Ionics as outlined in the Guide for Authors and in the Ethical Statement.

## Additional information

Correspondence and requests for materials should be addressed to AS.

## Funding

The financial support provided by The Research Fund of 10.13039/501100005790Faculty of Pharmacy, Thammasat University under The Specialized Research Grant, Research Unit: Medicinal Chemistry and Natural Products, Contact No. Pharm TU-S 1/2021 and AUDO.

## Declaration of competing interest

The authors declare that they have no known competing financial interests or personal relationships that could have appeared to influence the work reported in this paper.

## Data Availability

No data was used for the research described in the article.
