# Supplementary material for: Evaluation of Stemona collinsiae root extracts for topical cockroach control: adulticidal, nymphicidal, and chemical distribution analysis
Source: Toxicon X. 2025 May 9;26:100225. doi: 10.1016/j.toxcx.2025.100225 (PMC12149567; doi:10.1016/j.toxcx.2025.100225)
Supplement: Multimedia component 1 [file mmc1.docx]

**Supporting Information**

**Evaluation of *Stemona collinsiae* root extracts for topical cockroach control: adulticidal, nymphicidal, and chemical distribution analysis**

Aurapa Sakulpanich^1,*^, Anon Phayakkaphon^2^, Korawan Ounklong^3^, Jinnaphat Sommanat^3^, Yudthana Samung^2^, Raweewan Srisawat^2^, Jiraporn Ruangsittichai^2,**^

^1^ Division of Pharmaceutical Sciences, Faculty of Pharmacy, Thammasat University, Rangsit, Pathum Thani 12120, Thailand

^2^ Department of Medical Entomology, Faculty of Tropical Medicine, Mahidol University, Bangkok 10400, Thailand

^3^ National Science and Technology Development Agency, Thailand Science Park, Rangsit, Pathum Thani 12120, Thailand

*Corresponding author: Aurapa Sakulpanich^1^

**Co-corresponding author: Jiraporn Ruangsittichai^2^

^1^ Division of Pharmaceutical sciences, Faculty of Pharmacy, Thammasat University, Rangsit Campus, Pathum Thani 12120, Thailand https://orcid.org/0000-0001-5854-0462

E-mail: aurapa_s@tu.ac.th, aurapa.sak@gmail.com

^2^ Department of Medical Entomology, Faculty of Tropical Medicine, Mahidol University, Bangkok 10400, Thailand

E-mail: jiraporn.rua@mahidol.ac.th

**Content**

**Page**

| **Figure. S1** HPLC chromatograms of didehydrostemofoline appearing in the abdomen integument, lipid layer, alimentary canal and head extracts, after adult *P. americana* dropped with 1.5% w/v dichloromethane crude extract at 15 min | S2 |
| --- | --- |
| **Figure. S2** HPLC chromatograms of didehydrostemofoline appearing in the abdomen integument, lipid layer, alimentary canal and head extracts, after adult *P. americana* dropped with 1.5% w/v dichloromethane crude extract at 2 hours | S3 |
| **Figure. S3** HPLC chromatograms of didehydrostemofoline appearing in the abdomen integument, lipid layer, alimentary canal and head extracts, after adult *P. americana* dropped with 1.5% w/v dichloromethane crude extract at 24 hours | S4 |
| **Figure. S4** HPLC chromatograms of didehydrostemofoline appearing in the abdomen integument, lipid layer, alimentary canal and head extracts, after adult *P. americana* dropped with 10% w/v dichloromethane crude extract at 15 min | S5 |
| **Figure. S5** HPLC chromatograms of didehydrostemofoline appearing in the abdomen integument, lipid layer, alimentary canal and head extracts, after adult *P. americana* dropped with 10% w/v dichloromethane crude extract at 2 hours | S6 |
| **Figure. S6** HPLC chromatograms of didehydrostemofoline appearing in the abdomen integument, lipid layer, alimentary canal and head extracts, after adult *P. americana* dropped with 10% w/v dichloromethane crude extract at 24 hours | S7 |
| **Figure. S7** HPLC chromatograms of didehydrostemofoline appearing in the abdomen integument, lipid layer, alimentary canal and head extracts, after adult *P. americana* in negative control group receiving acetone only which did not appear a peak of didehydrostemofoline | S8 |


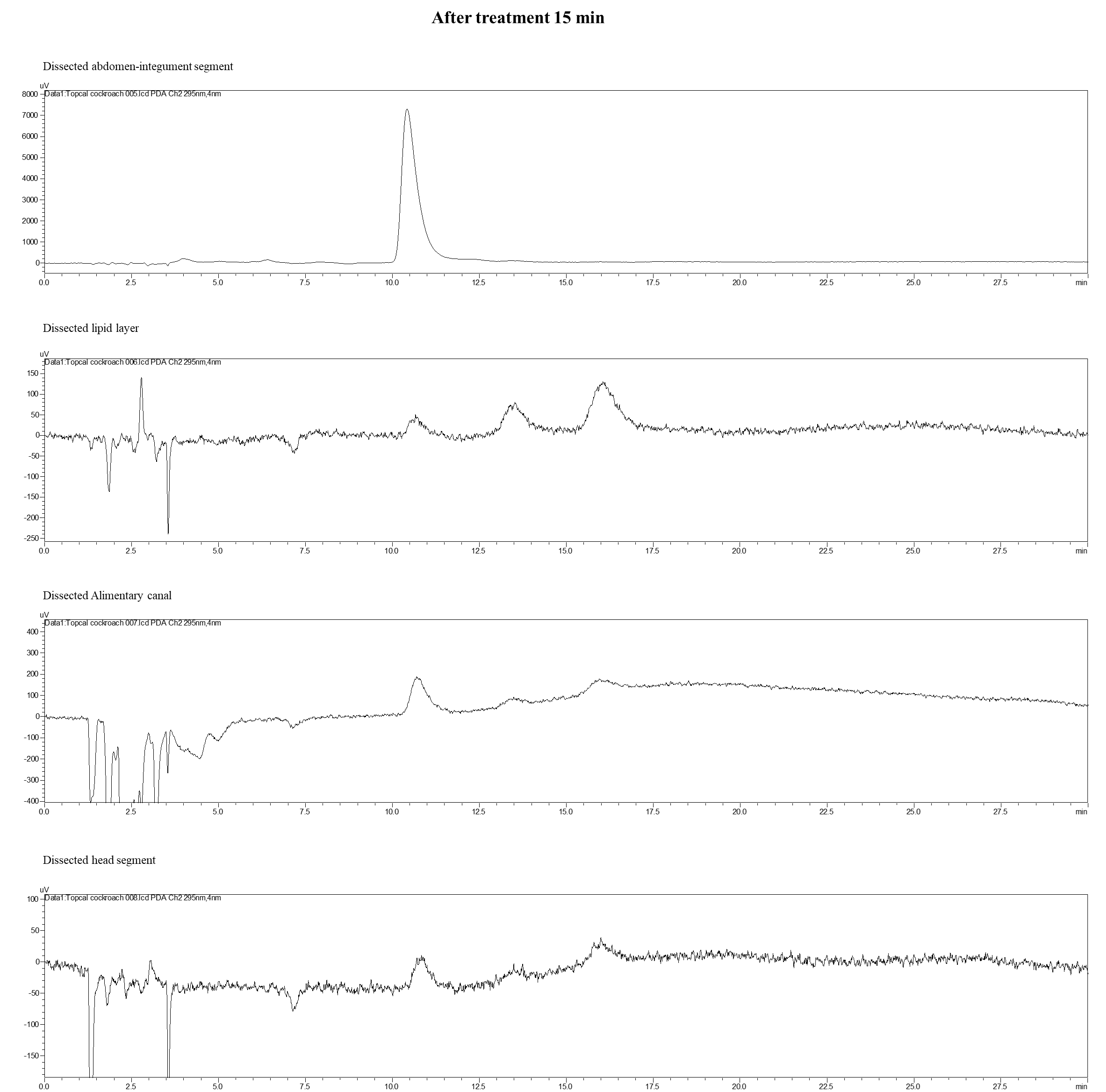


**Figure. S1** HPLC chromatograms of didehydrostemofoline appearing in the abdomen integument, lipid layer, alimentary canal and head extracts, after adult *P. americana* dropped with 1.5% w/v dichloromethane crude extract at 15 min


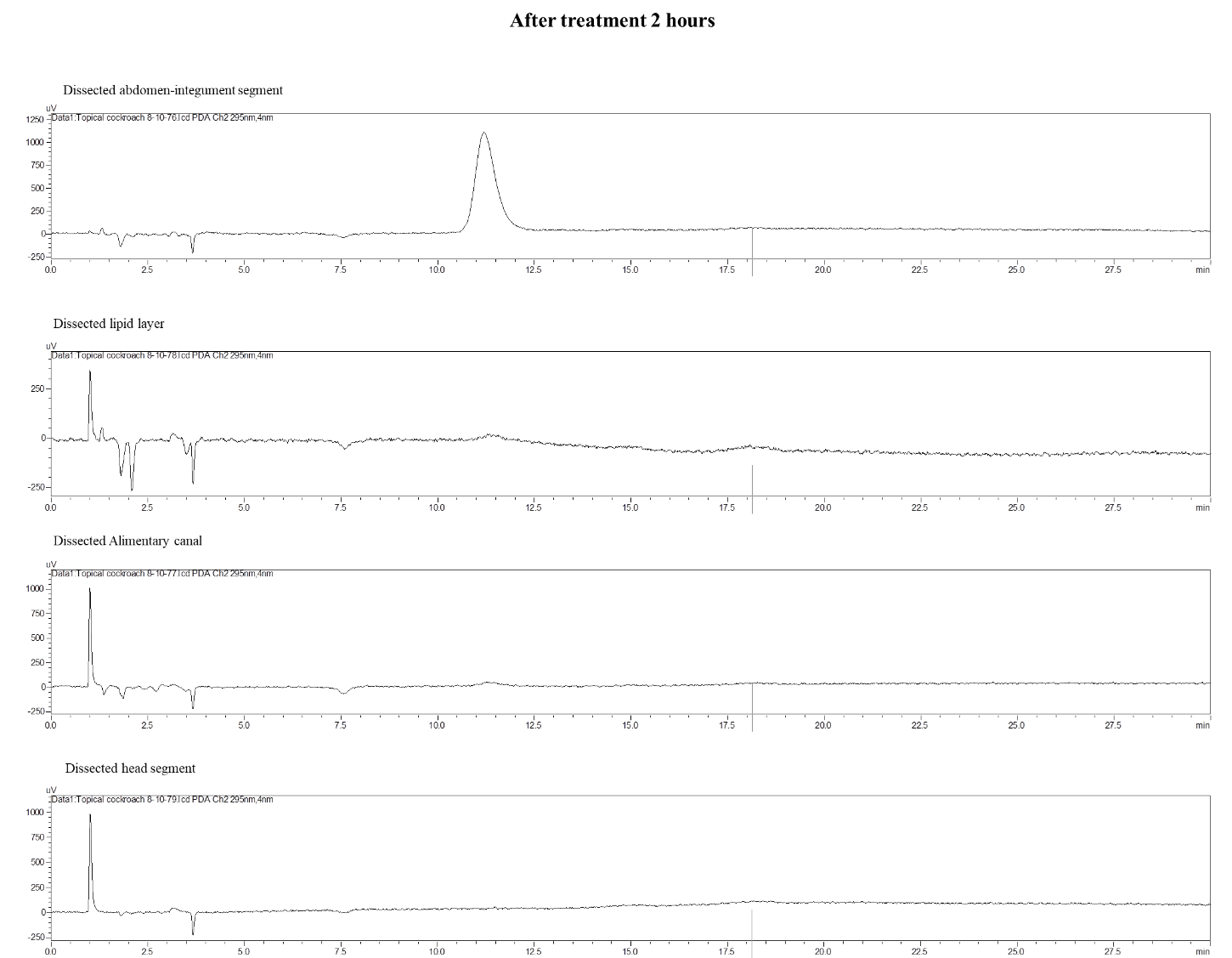


**Figure. S2** HPLC chromatograms of didehydrostemofoline appearing in the abdomen integument, lipid layer, alimentary canal and head extracts, after adult *P. americana* dropped with 1.5% w/v dichloromethane crude extract at 2 hours


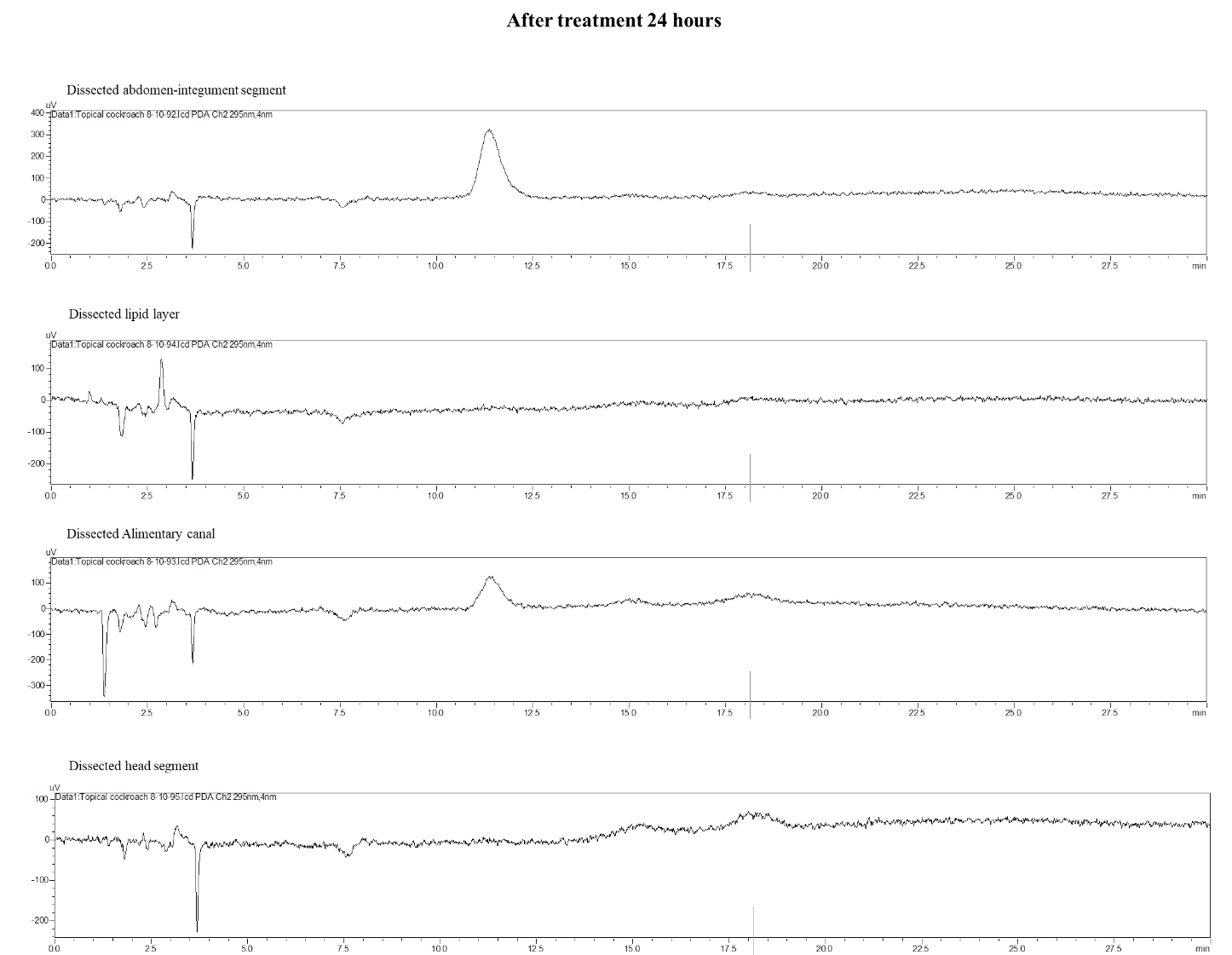


**Figure. S3** HPLC chromatograms of didehydrostemofoline appearing in the abdomen integument, lipid layer, alimentary canal and head extracts, after adult *P. americana* dropped with 1.5% w/v dichloromethane crude extract at 24 hours


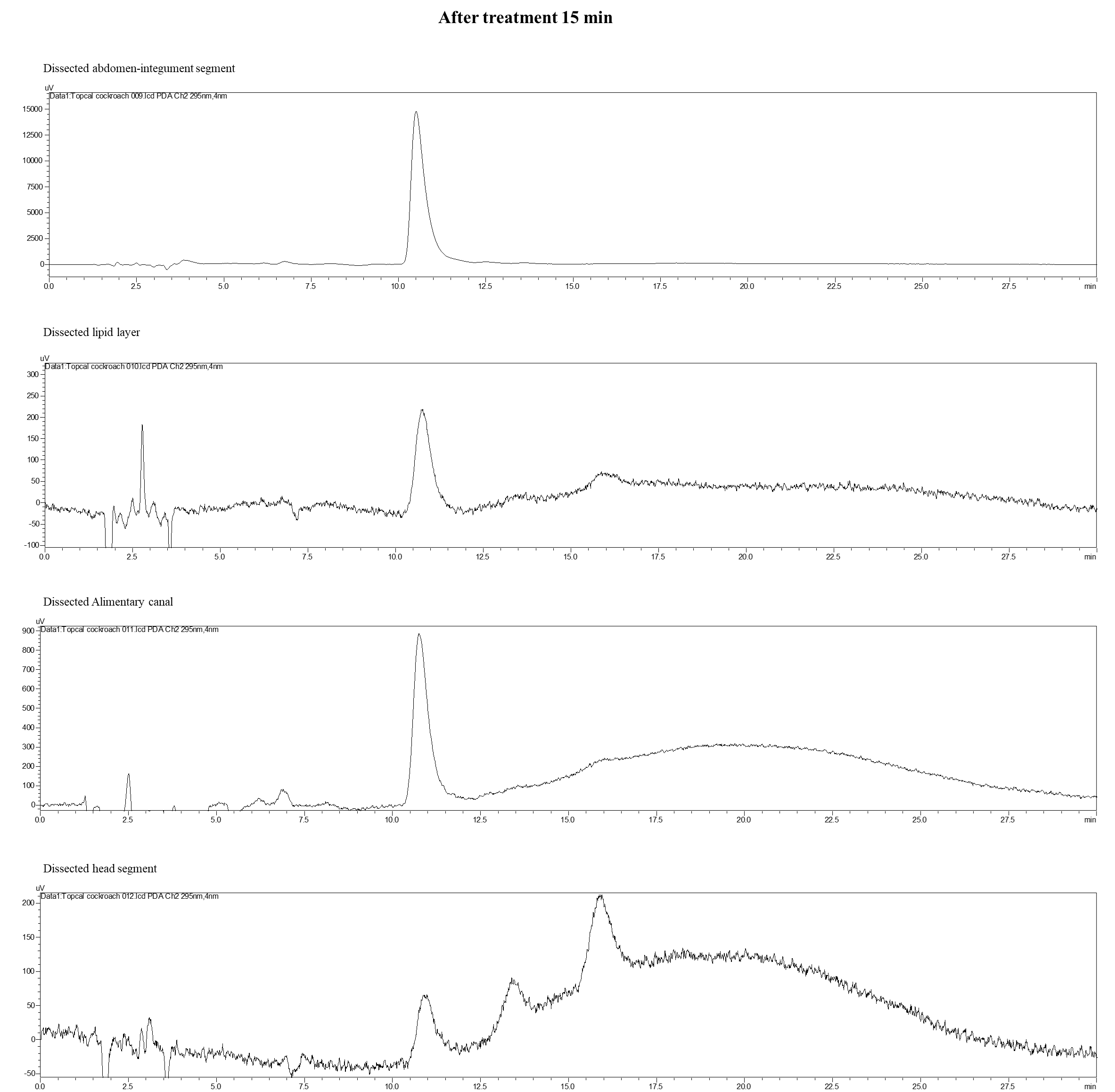


**Figure. S4** HPLC chromatograms of didehydrostemofoline appearing in the abdomen integument, lipid layer, alimentary canal and head extracts, after adult *P. americana* dropped with 10% w/v dichloromethane crude extract at 15 min


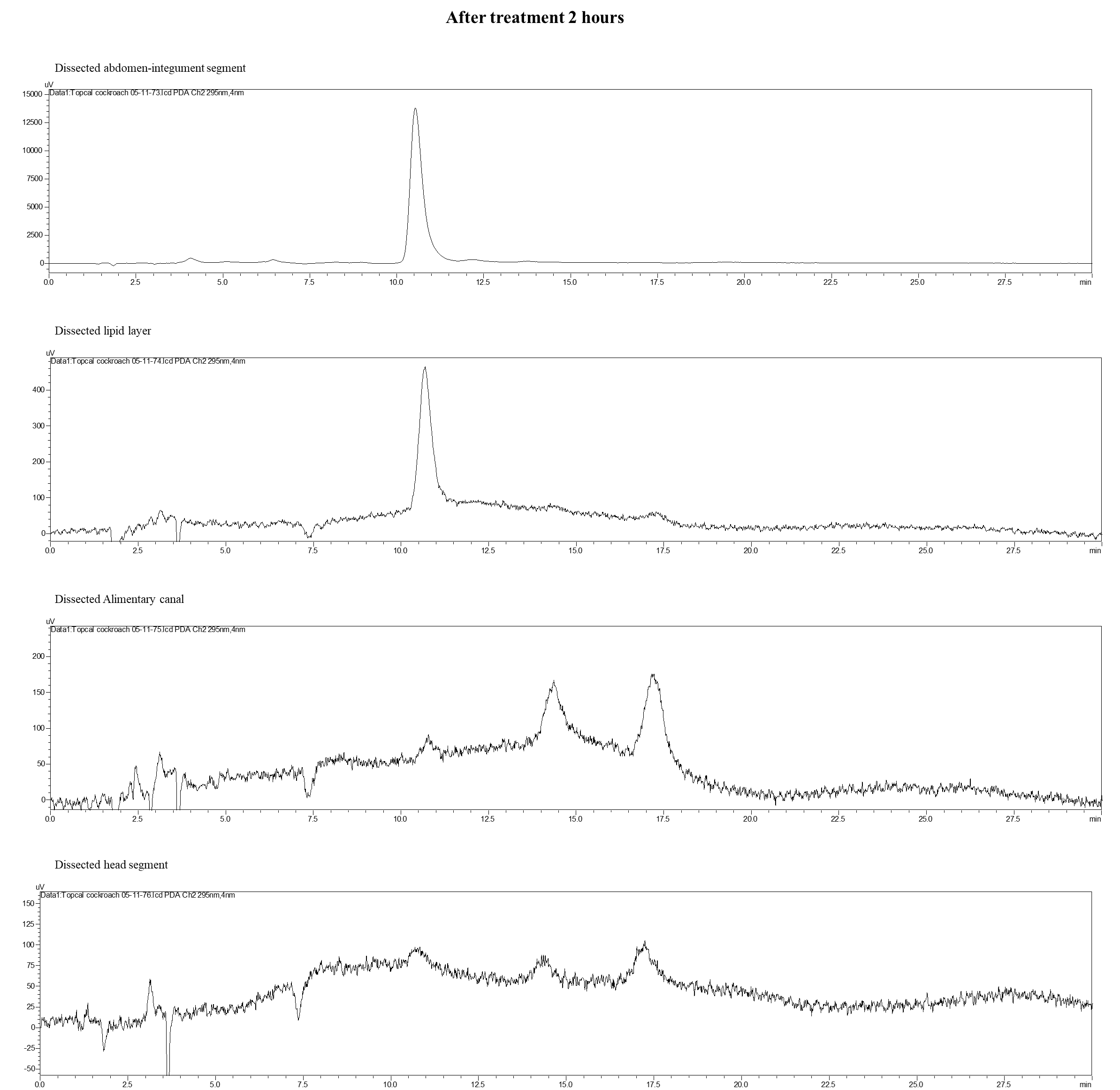


0

**Figure. S5** HPLC chromatograms of didehydrostemofoline appearing in the abdomen integument, lipid layer, alimentary canal and head extracts, after adult *P. americana* dropped with 10% w/v dichloromethane crude extract at 2 hours


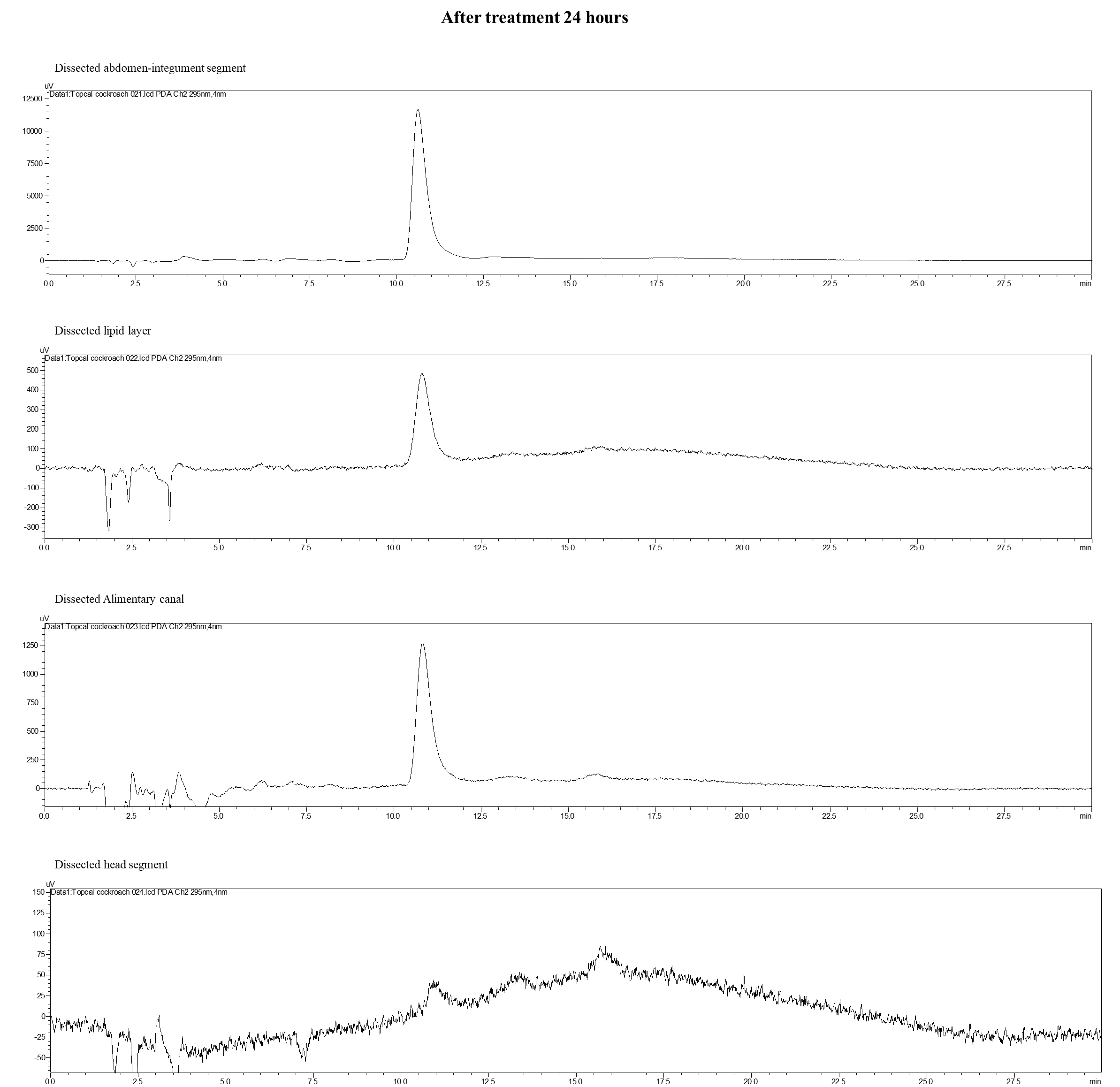


**Figure. S6** HPLC chromatograms of didehydrostemofoline appearing in the abdomen integument, lipid layer, alimentary canal and head extracts, after adult *P. americana* dropped with 10% w/v dichloromethane crude extract at 24 hours


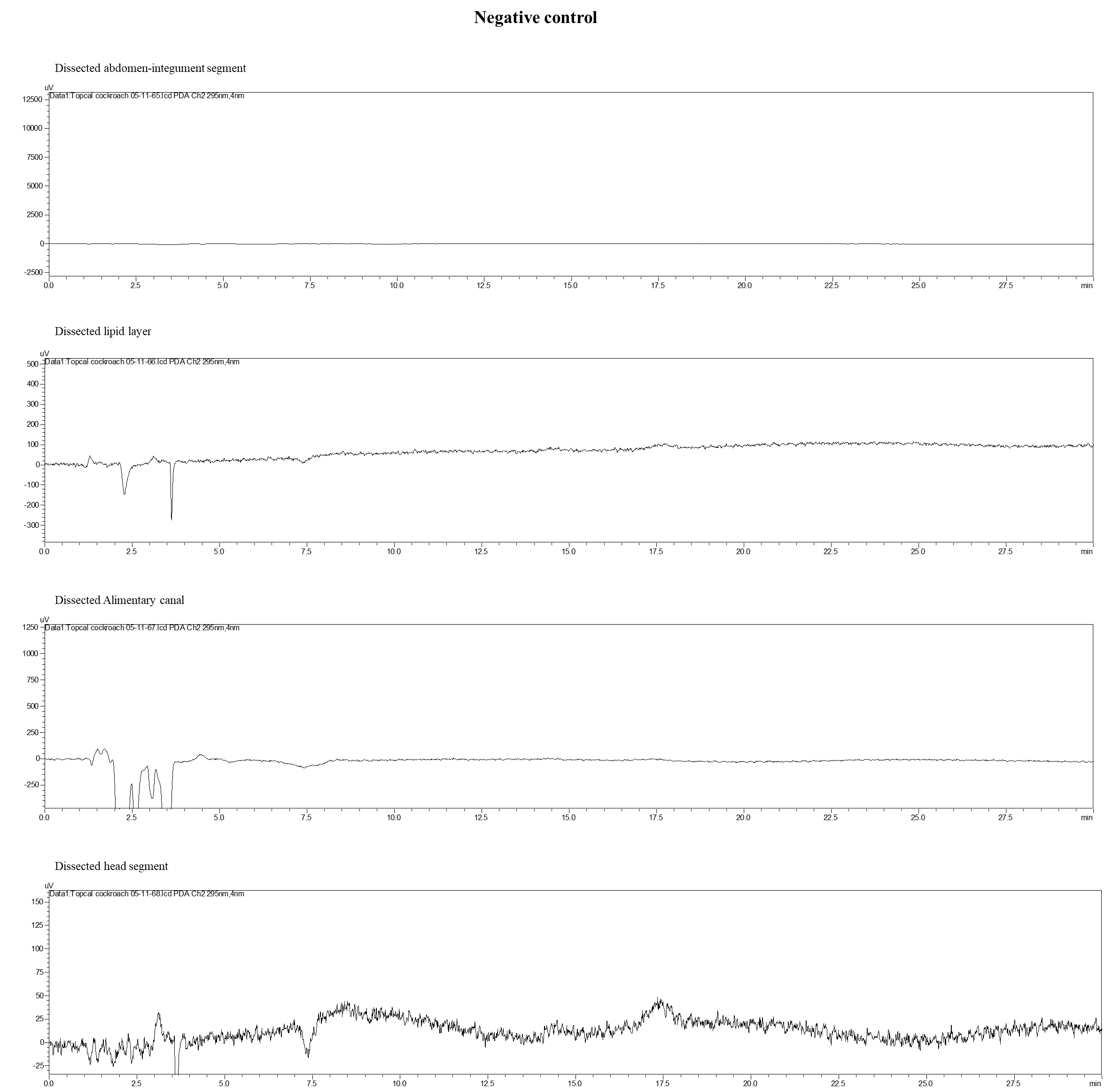


**Figure. S7** HPLC chromatograms of didehydrostemofoline appearing in the abdomen integument, lipid layer, alimentary canal and head extracts, after adult *P. americana* in negative control group receiving acetone only which did not appear a peak of didehydrostemofoline
